# Comparative effects of natural and functional lipid sources on physicochemical, textural, sensory, and nutritional lipid indices of spreadable processed cheese analogue

**DOI:** 10.1038/s41598-026-55188-3

**Published:** 2026-06-04

**Authors:** Shaimaa M. Hamdy, Amr A. Metwally, Mahmoud A. Deghedi, Hani S. Abdelmontaleb

**Affiliations:** Dairy Science Dept, Faculty of Agriculture, Fayoum University, Fayoum, Egypt

**Keywords:** Fatty acid profile, GC-MS, Health lipid indices, Multivariate analysis, PCA, Biochemistry, Health care, Plant sciences

## Abstract

This study evaluated the effects of natural and functional lipid sources, including butter, palm kernel oil, cocoa butter substitute, flaxseed oil, and sunflower oil, on the quality of spreadable processed cheese analogues during 60 days of refrigerated storage. Formulations were standardized to comparable moisture, fat, and protein contents to isolate the effect of lipid type on physicochemical, techno-functional, nutritional, and sensory properties. Lipid source significantly influenced meltability, oil separation, fatty acid composition, nutritional lipid indices, and sensory acceptability. Flaxseed oil markedly improved the lipid profile by reducing saturated fatty acids from 65.06% in the control to 14.98% and increasing unsaturated fatty acids to 85.02%, which reduced the atherogenic and thrombogenic indices from 2.14 to 2.87 to 0.13, respectively. However, this improvement was accompanied by greater oil separation and lower sensory scores, indicating reduced emulsion and sensory stability. Butter and sunflower oil formulations showed the highest sensory acceptability, with scores ranging from 8.1 to 8.9. Overall, the findings demonstrate that lipid selection strongly influences the balance between nutritional improvement, techno-functional stability, and consumer acceptability in spreadable processed cheese analogues.

## Introduction

Processed cheese analogue (PCA) constitutes a strategically important category of dairy-based formulated products designed to mimic the functional and sensory properties of traditional processed cheese while allowing flexibility in ingredient composition and cost optimization^1^. Among PCA variants, spreadable systems are particularly attractive due to their soft rheological profile, ease of application, and suitability for diverse food applications^2^. Unlike natural cheese, the structural integrity and performance of PCA are highly dependent on formulation design, particularly the characteristics of the incorporated lipid phase, which plays a central role in emulsion stabilization, matrix structuring, mouthfeel development, and thermal behavior^3^. In PCA systems, lipids function not only as flavor carriers but also as critical structuring agents that interact with hydrated proteins and emulsifying salts to form a stable fat-in-water emulsion matrix. The physicochemical performance of this matrix is strongly influenced by fatty acid composition, solid fat content (SFC), crystallization kinetics, and melting transitions^4^. Lipids rich in saturated fatty acids (SFAs) generally promote structural rigidity, improved oil binding, and enhanced emulsion stability, whereas highly unsaturated oils tend to reduce firmness and increase susceptibility to oil separation and oxidative degradation^5,6^. Achieving a balance between structural functionality and nutritional improvement therefore represents a major formulation challenge in spreadable PCA systems.

Traditionally, milk fat has served as the reference lipid phase in cheese products due to its desirable melting behavior and sensory contribution. However, its elevated SFA content and associated cardiovascular health concerns have stimulated research into alternative lipid systems^7^. In PCA manufacture, vegetable-based fats and structured lipid substitutes are increasingly utilized to modulate techno-functional properties while offering economic and compositional advantages^8,9^.

Palm kernel oil (PKO) is widely employed in cheese analogues because of its high lauric acid content and sharp melting transition, which contribute to firmness and polymorphic stability^10,11^. Similarly, cocoa butter substitutes (CBS), derived from fractionated and modified vegetable fats, provide tailored crystallization behavior and controlled melting characteristics suitable for textural optimization^12–14^. Nevertheless, both PKO and CBS remain predominantly saturated fat systems, limiting their nutritional improvement potential. Conversely, functional lipid sources such as flaxseed oil and sunflower oil present favorable fatty acid profiles. Flaxseed oil is exceptionally rich in α-linolenic acid (ω-3), whereas sunflower oil is characterized by high linoleic acid content, both associated with improved lipid health indices^15,16^. Despite their nutritional advantages, incorporation of highly unsaturated oils into PCA matrices poses technological challenges due to low solid fat content, weak crystallization capacity, and increased oxidative susceptibility^3,17^. Although numerous studies have evaluated partial fat replacement strategies in cheese systems, direct comparative investigations integrating natural saturated fats, structured substitutes, and nutritionally functional oils within a unified spreadable PCA model remain limited. Moreover, few studies have systematically linked fatty acid modifications to techno-functional behavior, sensory perception, and calculated lipid health indices (atherogenicity and thrombogenicity) within a single experimental framework.

Therefore, the present study aims to provide a comprehensive comparative evaluation of palm kernel oil, cocoa butter substitute, flaxseed oil, and sunflower oil as lipid phases in spreadable processed cheese analogue. Emphasis is placed on elucidating their effects on physicochemical stability, techno-functional properties, sensory quality, and lipid nutritional indices derived from fatty acid composition. By addressing the structural–nutritional trade-off inherent in lipid reformulation, this work contributes to the rational design of next-generation cheese analogue systems aligned with both technological performance requirements and contemporary nutritional expectations.

## Materials and methods

### Raw materials

Fresh buffalo skim milk (9.2% total solids; 3.4% protein, 4.9% lactose, 0.1% fat, 0.8% ash; 90.8% moisture) was purchased from the dairy herd of the Faculty of Agriculture, Fayoum University (Fayoum, Egypt). Skim milk powder (34% protein, 54% lactose, 5.75% ash, 5.0% moisture, 1.25% fat) was supplied by Arla Foods (Denmark/Sweden). Corn starch was procured locally (Giza, Egypt). A commercial emulsifier–stabilizer blend (CR15 grade), composed of mono- and diglycerides, plant-derived gums, and carrageenan, was used. Emulsifying salts (S20 and B3 grades; expressed as P_2_O_5_) were obtained from a local dairy ingredient supplier (Cairo, Egypt). Citric acid monohydrate, food-grade sodium chloride, and potassium sorbate were purchased from certified commercial suppliers. Cheddar and Roumy flavorings were incorporated to standardize sensory characteristics.

To assess the influence of lipid phase composition on processed cheese analogue performance, five fat sources were evaluated: unsalted sweet cream butter (82% fat), hydrogenated palm kernel oil (NCOTE 370 LT grade), cocoa butter substitute (vegetable-based structured fat), flaxseed oil, and sunflower oil. All lipid materials were food-grade and used as received. All ingredients were stored under manufacturer-recommended conditions prior to processing. The resulting spreadable processed cheese analogue samples were analyzed when fresh, and after 30 and 60 days of refrigerated storage at 4 ± 1 °C for physicochemical and functional properties. For clarity, unlike the other analyses, fatty acid profiling was performed as a single analytical determination per formulation.

## Methods

### Characterization of lipid sources

All lipid materials were characterized prior to formulation to evaluate their chemical quality, oxidative stability, and physicochemical properties relevant to processed cheese analogue performance. Analyses were conducted according to standard AOCS and AOAC methods unless otherwise specified^18] and [19^. Free fatty acid (FFA) content and acid value (AV) were determined using standard titrimetric procedures and expressed as percentage oleic acid and mg KOH/g fat, respectively, as indicators of hydrolytic degradation. Peroxide value (PV), reflecting primary lipid oxidation, was measured according to AOCS Cd 8–53 and expressed as milliequivalents of active oxygen per kilogram of fat (meq O_2_/kg). Iodine value (IV), determined according to AOCS official methods and calculated from fatty acid composition, was used to estimate the degree of unsaturation of each lipid source. Soap content was analyzed following AOCS Ca 17–91 to confirm the absence of residual alkaline impurities. Color was measured using a Lovibond Tintometer with a 1-inch optical cell and reported as Red/Yellow (R/Y) units where applicable. These parameters were considered critical for predicting lipid crystallization behavior, oxidative susceptibility, and structural functionality within the emulsified cheese analogue matrix.

### Processing of spreadable processed cheese analogue

Spreadable processed cheese analogue samples were manufactured according to a standardized pilot-scale protocol to ensure consistency among treatments^2^. Processing was performed using a laboratory cheese cooker equipped with mechanical agitation and temperature control. Fresh buffalo skim milk was preheated to 60 °C, after which skim milk powder and corn starch were gradually incorporated under continuous agitation to form a homogeneous aqueous–protein phase. The designated lipid source for each treatment was subsequently added and dispersed to form a preliminary fat-in-water emulsion. Emulsifying salts, citric acid, sodium chloride, potassium sorbate, flavoring agents, and the stabilizer–emulsifier blend were sequentially incorporated under controlled mixing.

The blend (10 kg batch each) was heated to 90 °C and maintained for 15 min with continuous agitation to ensure complete lipid melting, calcium sequestration by emulsifying salts, casein hydration, and formation of a stable protein-stabilized emulsion matrix. The resulting smooth and cohesive mass was hot-filled into pre-sterilized containers, cooled to ambient temperature, and stored at 4 ± 1 °C. Formulations for each treatment are presented in Table 1.

**Table 1:** Experimental formulations of spreadable processed cheese analogue.

Ingredients (g)	C	T1	T2	T3	T4	T5
Fresh cow milk	558	618	618	618	618	618
Skim milk powder	55	50	50	50	50	50
Corn starch	30	30	30	30	30	30
CR_15_ (Stabilizer)	12	12	12	12	12	12
Emulsifying salts (S20 + B3)	12	12	12	12	12	12
Citric acid	1.5	1.5	1.5	1.5	1.5	1.5
Salt (NaCl)	7	7	7	7	7	7
Potassium sorbate	1	1	1	1	1	1
Cheddar, Romy cheese flavor	6	6	6	6	6	6
Lipid source	305 (Butter)	250 (PKO)	250 (CBS)	125 PKO + 125 CBS	250 (Flaxseed)	250 (Sunflower)
Water	12.5	12.5	12.5	12.5	12.5	12.5

All ingredients are expressed in grams, corresponding to 1 kg of each spreadable processed cheese formulation. C= spreadable cheese analogue with butter, T1 = spreadable cheese analogue with palm kernel oil, T2 = spreadable cheese analogue with cocoa butter substitute, T3 = spreadable cheese analogue with 50%palm kernel oil + 50% cocoa butter substitute, T4 = spreadable cheese analogue with flaxseed oil, T5 = spreadable cheese analogue with sunflower oil.

### Chemical composition of spreadable processed cheese analogue

All spreadable processed cheese analogue samples were analyzed in triplicate to determine their physicochemical composition. The pH was measured at 25 °C using a calibrated digital pH meter (Kent EIL 7020, UK). Titratable acidity was determined according to^19^, and expressed as percentage lactic acid. Proximate composition, including moisture, fat, protein, and ash contents, was determined in accordance with^19^. Moisture content was measured by oven-drying to constant weight, fat content by the standard extraction method, protein by the Kjeldahl method, and ash by incineration in a muffle furnace. All measurements were performed in triplicate, and results were expressed on a percentage basis.

### Texture profile analysis

Texture profile analysis (TPA) was performed using a texture analyzer (TA.XTplus, Stable Micro Systems, UK) equipped with a 36 mm diameter cylindrical aluminum probe (P/36R). Prior to testing, samples were equilibrated at 25 ± 1 °C and prepared in cylindrical molds (20 mm height × 25 mm diameter) to ensure uniform geometry. Samples were subjected to a double-compression cycle to 30% strain of their original height, simulating two consecutive bites. The pre-test, test, and post-test speeds were set at 1.0 mm/s, with a trigger force of 5 g. A 5 s interval was maintained between compressions. Force–time curves were recorded, and textural parameters including hardness, springiness, cohesiveness, gumminess, chewiness, and adhesiveness were calculated automatically using the instrument software (Exponent, Stable Micro Systems). TPA was performed following standard procedures described by Bourne^20^ and adapted for processed cheese systems^3^.

### Color measurement

Color parameters were determined using a calibrated instrumental colorimeter operating within the CIELAB color space (CIE, 1976). Prior to analysis, samples were equilibrated at 25 ± 1 °C and their surfaces were carefully leveled to ensure uniform reflectance. The instrument was calibrated against a standard white reference tile before measurement. Color coordinates were recorded using illuminant D65 and a 2° standard observer. The parameters L* (lightness), a* (red–green axis), and b* (yellow–blue axis) were measured at three randomly selected surface points per sample, and mean values were calculated. The Whiteness Index (WI) was calculated from L*, a*, and b* values according to the CIE method, while the Yellowness Index (YI) was determined based on ASTM D1925 standard procedures^21^, under identical measurement conditions^13^.

### Lipid extraction

Before lipid extraction, processed cheese samples were equilibrated to refrigeration temperature and finely ground using an analytical mill (A11 basic, IKA, Germany). Total lipids were extracted using a modified Folch procedure according to^22^. In brief, 4 g of the homogenized sample was combined with 40 mL of chloroform–methanol (2:1, v/v) and subjected to high-shear homogenization using an Ultra-Turrax T25 (IKA, Staufen, Germany) to ensure complete matrix disruption and lipid release. The homogenate was subsequently agitated for 30 min in a temperature-controlled thermomixer (Eppendorf AG, Hamburg, Germany) to enhance lipid solubilization. The mixture was then filtered through qualitative filter paper (Ø 185 mm; Macherey-Nagel, Düren, Germany) into a separatory funnel. Phase separation was achieved by adding 10 mL of brine, allowing clear separation of the organic layer. The chloroform phase containing the extracted lipids was collected, dried over anhydrous sodium sulfate to remove residual moisture, and concentrated to dryness under reduced pressure. The recovered lipid fraction was flushed with nitrogen and stored at − 20 °C until subsequent analyses.

### Preparation of fatty acid methyl esters

Extracted lipids were derivatized to fatty acid methyl esters (FAMEs) using a base-catalyzed transesterification method. Briefly, lipid samples were transferred into Teflon-lined screw-cap reaction tubes, and approximately 6 mL of 0.5 N sodium methoxide solution was added to each tube. The transesterification reaction was conducted at 50 °C for 10 min to ensure efficient methylation of fatty acids, following the method described^23^. Upon completion of the reaction, the tubes were allowed to cool to room temperature, after which 2 mL of isooctane and 3 mL of 10% (v/v) acetic acid were added to quench the reaction and promote phase separation. To minimize the loss of volatile short-chain fatty acids, the tubes were immediately sealed. The resulting mixture was centrifuged at 2000 × g for 10 min to achieve clear phase separation. The upper isooctane layer containing the FAMEs was carefully collected, transferred into gas-tight chromatography vials, and stored at − 20 °C until gas chromatographic analysis.

### Fatty acids profile

The fatty acid composition of the processed cheese samples was determined by gas chromatography–mass spectrometry (GC–MS) following the method described by^24^. Analyses were performed using a TRACE Ultra gas chromatograph (Thermo Scientific, Santa Clara, CA, USA) coupled to an ISQ single quadrupole mass spectrometer. Helium was used as the carrier gas at a constant flow rate of 1.0 mL/min, with a split injection ratio of 1:10. Chromatographic separation was achieved using an oven temperature program that commenced at 60 °C (held for 1 min), followed by a linear ramp of 4 °C/min to 240 °C, which was maintained for an additional 1 min. The injector and MS transfer line temperatures were both set at 210 °C. Prior to analysis, FAME samples were diluted in hexane at a ratio of 1:10 (v/v), and 1 µL of the diluted solution was injected for each run. The total chromatographic run time was approximately 10 min. Mass spectra were acquired under electron ionization (EI) conditions at 70 eV, scanning a mass-to-charge (m/z) range of 40–450. Fatty acids were identified using Automated Mass Spectral Deconvolution and Identification System (AMDIS) software through spectral deconvolution and comparison with reference mass spectral libraries. Identification was further confirmed by calculating retention indices relative to a homologous series of n-alkanes (C6–C22), ensuring accurate and reliable compound assignment.

### Reagents, quality control and method validation

All reagents, solvents, and standards used for lipid extraction, FAME preparation, and GC–MS analysis were of analytical or chromatographic grade and obtained from certified commercial suppliers. Chloroform (≥ 99.8%), methanol (≥ 99.9%), hexane (≥ 99%), isooctane (≥ 99%), acetic acid (glacial, ≥ 99.7%), sodium methoxide solution (0.5 N in methanol), and anhydrous sodium sulfate were purchased from Merck (Sigma-Aldrich, Darmstadt, Germany). Deionized water was produced using a laboratory water purification system meeting analytical-grade specifications. Certified FAME reference standards and a homologous series of n-alkanes (C6–C22), used for compound identification and retention index determination, were obtained from Supelco (Sigma-Aldrich, Bellefonte, PA, USA). All standards were stored under refrigerated conditions and protected from light according to the manufacturers’ recommendations.

GC–MS analyses were performed using validated analytical procedures in accordance with ISO/IEC 17,025 quality assurance principles. Instrument performance was verified daily through mass axis tuning and sensitivity checks using manufacturer-recommended calibration compounds. System suitability testing was conducted at the beginning of each analytical batch by assessing retention time stability, chromatographic resolution, peak symmetry, and signal-to-noise ratios using certified reference standards. Method linearity was evaluated using multi-point calibration curves prepared from certified FAME standards over concentration ranges relevant to the expected fatty acid levels in processed cheese samples. Calibration curves demonstrated excellent linearity, with coefficients of determination (R²) ≥ 0.995 for all quantified fatty acids. Quantification was based on peak area responses, and calibration verification standards were analyzed intermittently throughout each analytical sequence to ensure instrumental stability. Limits of detection (LOD) and limits of quantification (LOQ) were determined based on signal-to-noise ratios of 3:1 and 10:1, respectively. LOD values ranged from approximately 0.01 to 0.05 mg/g fat, while LOQ values ranged from approximately 0.03 to 0.15 mg/g fat, depending on fatty acid chain length and degree of unsaturation. Quality control procedures included the analysis of procedural blanks, solvent blanks, and replicate samples to monitor contamination, carryover, and analytical precision. Method repeatability was assessed by repeated injections and independent sample preparations, with relative standard deviations (RSDs) maintained below 5% for major fatty acids. Fatty acid identification was confirmed through mass spectral matching against validated libraries and comparison of calculated retention indices with those of reference standards. All reagents and standards were stored under controlled conditions in accordance with the manufacturers’ specifications, and expiration dates were strictly observed. Glassware and consumables were prepared following documented laboratory cleaning and handling procedures to minimize contamination. All chromatographic data, calibration records, and quality control results were archived to ensure full traceability and data integrity prior to statistical analysis.

### Lipid quality indices

The nutritional and health-related quality of the processed cheese samples was evaluated through a comprehensive assessment of their fatty acid composition. Lipid quality indices were calculated to characterize the balance between saturated and unsaturated fatty acids and to estimate the potential implications of lipid intake on cardiovascular health. Based on the determined fatty acid profiles, the following compositional parameters were calculated: total saturated fatty acids (ΣSFA), total unsaturated fatty acids (ΣUFA), total monounsaturated fatty acids (ΣMUFA), total polyunsaturated fatty acids (ΣPUFA), unsaturated-to-saturated fatty acid ratio (UFA/SFA), PUFA-to-MUFA ratio (PUFA/MUFA), PUFA-to-SFA ratio (PUFA/SFA), linoleic acid to α-linolenic acid ratio (LA/ALA), and total trans fatty acids (TFA).

In addition to these compositional ratios, several established health-related lipid indices were computed to provide deeper insight into the nutritional quality and potential cardiometabolic impact of the lipid fractions. These indices included desirable fatty acids (DFA), atherogenic index (AI), thrombogenic index (TI), health-promoting index (HPI), hypocholesterolemic to hypercholesterolemic fatty acid ratio (H/H), unsaturation index (UI), hypercholesterolemic saturated fatty acids (HFSA), nutritive value index (NVI), and lipid preventive score (LPS). The nutritive value index (NVI) was calculated according to the method described by^25^. All equations used for the calculation of lipid quality indices were adopted from previously published models reported by^26^. The specific mathematical expressions applied in this study are presented below and are numbered sequentially from following equations:

$$\:\mathrm{D}\mathrm{F}\mathrm{A}=\mathrm{\%}\:\mathrm{C}18:0+\mathrm{\%}\:\mathrm{M}\mathrm{U}\mathrm{F}\mathrm{A}+\mathrm{\%}\:\mathrm{P}\mathrm{U}\mathrm{F}\mathrm{A}$$

$$\:\mathrm{A}\mathrm{I}=\frac{(\mathrm{C}12:0\:+\:4\times\:\mathrm{C}14:0\:+\:\mathrm{C}16:0)}{({\upomega\:}-3\:\mathrm{P}\mathrm{U}\mathrm{F}\mathrm{A}\:+\:{\upomega\:}-6\:\mathrm{P}\mathrm{U}\mathrm{F}\mathrm{A}\:+\:\mathrm{M}\mathrm{U}\mathrm{F}\mathrm{A})}$$

$$\:\mathrm{T}\mathrm{I}=\frac{(\mathrm{C}14:0\:+\:\mathrm{C}16:0\:+\:\mathrm{C}18:0)}{(0.5\mathrm{M}\mathrm{U}\mathrm{F}\mathrm{A}\:+\:0.5\:{\upomega\:}-6\:\mathrm{P}\mathrm{U}\mathrm{F}\mathrm{A}\:+\:3\:{\upomega\:}-3\mathrm{P}\mathrm{U}\mathrm{F}\mathrm{A}\:+\:{\upomega\:}-3/{\upomega\:}-6)}$$

$$\:\mathrm{H}\mathrm{P}\mathrm{I}=\frac{{\Sigma\:}\mathrm{U}\mathrm{F}\mathrm{A}}{[\mathrm{C}12:0+(4\:\mathrm{x}\:\mathrm{C}14:0)\:+\:\mathrm{C}16:0]}$$

$$\:\mathrm{H}\mathrm{H}=\frac{(\mathrm{c}\mathrm{i}\mathrm{s}-\mathrm{C}18:1\:+\:{\Sigma\:}\mathrm{P}\mathrm{U}\mathrm{F}\mathrm{A})}{\left(\mathrm{C}12:0\:+\:\mathrm{C}14:0+\:\mathrm{C}16:0\right)}$$

$$\:\mathrm{U}\mathrm{I}=1\:\mathrm{x}\:\left(\mathrm{\%}\mathrm{m}\mathrm{o}\mathrm{n}\mathrm{o}\mathrm{e}\mathrm{n}\mathrm{o}\mathrm{i}\mathrm{c}\mathrm{s}\right)+2\:\mathrm{x}\:\left(\mathrm{\%}\mathrm{d}\mathrm{i}\mathrm{e}\mathrm{n}\mathrm{o}\mathrm{i}\mathrm{c}\mathrm{s}\right)+3\:\mathrm{x}\:\left(\mathrm{\%}\mathrm{t}\mathrm{r}\mathrm{i}\mathrm{e}\mathrm{n}\mathrm{o}\mathrm{i}\mathrm{c}\mathrm{s}\right)+4\:\mathrm{x}\:\left(\mathrm{\%}\mathrm{t}\mathrm{e}\mathrm{t}\mathrm{r}\mathrm{a}\mathrm{e}\mathrm{n}\mathrm{o}\mathrm{i}\mathrm{c}\mathrm{s}\right)+5\:\mathrm{x}\:\left(\mathrm{\%}\mathrm{p}\mathrm{e}\mathrm{n}\mathrm{t}\mathrm{e}\mathrm{n}\mathrm{o}\mathrm{i}\mathrm{c}\mathrm{s}\right)+6\:\mathrm{x}\:\left(\mathrm{\%}\mathrm{h}\mathrm{e}\mathrm{x}\mathrm{a}\mathrm{e}\mathrm{n}\mathrm{o}\mathrm{i}\mathrm{c}\mathrm{s}\right)$$

$$\:\frac{\mathrm{L}\mathrm{A}}{\mathrm{A}\mathrm{L}\mathrm{A}}=\:\frac{\mathrm{C}18:2\:{\upomega\:}-6\:\:\left(\mathrm{L}\mathrm{i}\mathrm{n}\mathrm{o}\mathrm{l}\mathrm{e}\mathrm{i}\mathrm{c}\:\mathrm{a}\mathrm{c}\mathrm{i}\mathrm{d}\right)}{\mathrm{C}18:3\:{\upomega\:}-3\:({\upalpha\:}-\mathrm{L}\mathrm{i}\mathrm{n}\mathrm{o}\mathrm{l}\mathrm{e}\mathrm{n}\mathrm{i}\mathrm{c}\:\mathrm{a}\mathrm{c}\mathrm{i}\mathrm{d})}$$

$$\:\mathrm{T}\mathrm{F}\mathrm{A}={\Sigma\:}\mathrm{T}\mathrm{F}\mathrm{A}$$

$$\:\mathrm{H}\mathrm{S}\mathrm{F}\mathrm{A}=\mathrm{C}12:0+\mathrm{C}14:0+\mathrm{C}16:0$$

$$\:\mathrm{N}\mathrm{V}\mathrm{I}=\frac{(\mathrm{C}18:0+\mathrm{C}18:1)}{\mathrm{C}16:0}$$

$$\:\mathrm{L}\mathrm{P}\mathrm{S}=\mathrm{F}\mathrm{A}\mathrm{T}+2\times\:\mathrm{S}\mathrm{F}\mathrm{A}-\mathrm{M}\mathrm{U}\mathrm{F}\mathrm{A}- {\rm 0.5PUFA}$$

### Functional characteristics

The meltability of the processed cheese samples was evaluated using a modified Schreiber method. For each treatment, three cheese discs of uniform dimensions (4 cm in diameter and 10 mm in thickness) were prepared and placed individually in glass Petri dishes as described by^3^. The samples were heated in a forced-air oven at 110 °C for 10 min to induce melting, then allowed to cool at ambient temperature for 15 min. Meltability was expressed as the percentage increase in surface area relative to the original disc area, calculated using the following equation:

$$\:\mathrm{M}\mathrm{e}\mathrm{l}\mathrm{t}\mathrm{a}\mathrm{b}\mathrm{i}\mathrm{l}\mathrm{i}\mathrm{t}\mathrm{y}=\frac{\mathrm{m}\mathrm{e}\mathrm{l}\mathrm{t}\mathrm{e}\mathrm{d}\:\mathrm{a}\mathrm{r}\mathrm{e}\mathrm{a}-\mathrm{o}\mathrm{r}\mathrm{i}\mathrm{g}\mathrm{i}\mathrm{n}\mathrm{a}\mathrm{l}\:\mathrm{a}\mathrm{r}\mathrm{e}\mathrm{a}}{\mathrm{o}\mathrm{r}\mathrm{i}\mathrm{g}\mathrm{i}\mathrm{n}\mathrm{a}\mathrm{l}\:\mathrm{a}\mathrm{r}\mathrm{e}\mathrm{a}\:}\:\mathrm{x}\:100$$

Oil separation was determined according to the method described by^27^. Briefly, three discs from each treatment were placed on Whatman No. 41 filter paper and heated at 110 °C for 10 min. During heating, released fat migrated into the surrounding filter paper, forming a visible grease halo around each disc. The extent of oil separation was quantified by calculating the relative increase in area attributable to fat diffusion, using the following equation:

$$\:\mathrm{O}\mathrm{i}\mathrm{l}\:\mathrm{s}\mathrm{e}\mathrm{p}\mathrm{a}\mathrm{r}\mathrm{a}\mathrm{t}\mathrm{i}\mathrm{o}\mathrm{n}=\frac{\mathrm{a}\mathrm{r}\mathrm{e}\mathrm{a}\:\mathrm{o}\mathrm{f}\:\mathrm{f}\mathrm{a}\mathrm{t}\:\mathrm{r}\mathrm{i}\mathrm{n}\mathrm{g}\:\mathrm{a}\mathrm{n}\mathrm{d}\:\mathrm{d}\mathrm{i}\mathrm{s}\mathrm{c}-\mathrm{o}\mathrm{r}\mathrm{i}\mathrm{g}\mathrm{i}\mathrm{n}\mathrm{a}\mathrm{l}\:\mathrm{d}\mathrm{i}\mathrm{s}\mathrm{c}\:\mathrm{a}\mathrm{r}\mathrm{e}\mathrm{a}}{\mathrm{o}\mathrm{r}\mathrm{i}\mathrm{g}\mathrm{i}\mathrm{n}\mathrm{a}\mathrm{l}\:\mathrm{d}\mathrm{i}\mathrm{s}\mathrm{c}\:\mathrm{a}\mathrm{r}\mathrm{e}\mathrm{a}\:}\:\mathrm{x}\:100$$

All meltability and oil separation measurements were performed in triplicate, and results are reported as mean values for each treatment.

### Sensory evaluation

Sensory evaluation of the processed cheese samples was conducted at room temperature across three storage intervals: fresh, one month and after two months. The assessment focused on five key sensory attributes: appearance, texture, flavor, aroma, and overall acceptability. A panel of 20 participants (12 males and 8 females) aged between 20 and 45 years was recruited, consisting of university faculty members and postgraduate students. While the panelists were not formally trained in sensory analysis, they were regular consumers of processed cheese (at least three times per week) and had familiarity with dairy products, particularly cheese quality characteristics. Samples were served in neutral white plastic cups under natural light to minimize visual bias. The evaluation employed a nine-point hedonic scale ranging from 1 (dislike extremely) to 9 (like extremely), following the method outlined by^3^. To ensure palate neutrality between samples, participants rinsed their mouths with water after tasting each sample. Overall acceptability scores for each cheese variant were calculated by averaging the individual ratings across all sensory attributes at each storage period. All sensory evaluation procedures were conducted in accordance with the guidelines of the American Dairy Science Association and Egyptian national regulations governing research involving human participants. The experimental protocol was reviewed and approved by the Research Ethics Committee of the Faculty of Agriculture, Fayoum University, Egypt. All participants were adults and provided written informed consent prior to participation.

### Statistical analysis

All experiments were conducted in triplicate, and results are presented as mean ± standard deviation. Data were analyzed using two-way analysis of variance (ANOVA) to assess the main effects of lipid source on physicochemical properties, fatty acid composition, techno-functional parameters, and sensory attributes of spreadable processed cheese analogue samples. When significant differences were observed, means were separated using Tukey’s honestly significant difference (HSD) test. Statistical significance was established at *p* < 0.05. To explore multivariate relationships among variables, principal component analysis (PCA) was performed on autoscaled data to evaluate sample discrimination and identify key contributors to variation. Hierarchical cluster analysis (HCA) was conducted using Ward’s linkage method with Euclidean distance to assess sample grouping patterns. Pearson correlation coefficients were calculated to examine associations among compositional, functional, and nutritional lipid indices. Multivariate analyses were performed using MATLAB R2012b (The MathWorks, Natick, MA, USA) with PLS Toolbox (version 7.5; Eigenvector Research, USA). Additional statistical processing and graphical visualization were carried out using Python (v3.9) with the Pandas, NumPy, SciPy, Matplotlib, and Seaborn libraries.

## Results and discussion

### Characterization of lipid sources

The physicochemical properties of the lipid materials as shown in Table 2 revealed clear compositional and stability differences between predominantly saturated fats (palm kernel oil and cocoa butter substitute) and highly unsaturated functional oils (flaxseed and sunflower oils). These variations are expected to directly influence oxidative stability, fat crystallization behavior, emulsion stability, and structural performance in spreadable processed cheese analogue systems.

**Table 2:** Physicochemical properties of lipid sources used in spreadable processed cheese analogue.

Characteristics	Lipid sources			
	Palm kernel oil (PKO)	Cocoa butter substitute (CBS)	Flaxseed oil	Sunflower oil
**Peroxide value (meq O₂/kg)**	1.73	0.10	3.34	5.15
**Iodine value (g I₂/100 g)**	18	37.5	187.5	125
**Free fatty acids (FFA%)**	0.11	0.13	1.07	0.07
**Acid value (mg KOH/g)**	0.218	0.258	2.128	0.139
**Soap content (%)**	0.00	0.00	0.00	0.00
**Color (Red/Yellow units)**	-	-	6.5/35 R/Y	0.8/10 R/Y

PV, an indicator of primary lipid oxidation, differed substantially among the oils. Cocoa butter substitute exhibited the lowest PV (0.10 meq O_2_/kg), followed by palm kernel oil (1.73 meq O_2_/kg), reflecting their high saturated fatty acid content and inherent oxidative resistance^28^. The absence of double bonds in saturated fatty acids limits hydroperoxide formation, enhancing thermal and storage stability. In contrast, flaxseed oil (3.34 meq O_2_/kg) and sunflower oil (5.15 meq O_2_/kg) displayed higher PV values, consistent with their elevated PUFA content^29^. Flaxseed oil, rich in α-linolenic acid, is particularly susceptible to oxidative degradation, suggesting that antioxidant strategies may be required when incorporating such oils into PCA formulations to prevent off-flavor development and quality deterioration^30,31^.

IV further confirmed marked differences in unsaturation. Palm kernel oil exhibited a low IV (18 g I_2_/100 g), characteristic of highly saturated fats capable of forming rigid crystalline networks that contribute to structural firmness in cheese analogues. Cocoa butter substitute showed moderate unsaturation (37.5 g I_2_/100 g), consistent with its semi-solid behavior and plasticity. In contrast, flaxseed (187.5 g I_2_/100 g) and sunflower oils (125 g I_2_/100 g) demonstrated high IV values, indicative of soft lipid phases with limited crystallization capacity. While such oils improve the nutritional fatty acid profile by increasing unsaturated fatty acid content, their low solid fat content may weaken the fat–protein matrix, increasing the risk of oil separation in spreadable systems. FFA content and AV provided additional indicators of hydrolytic stability. Flaxseed oil exhibited markedly higher FFA (1.07%) and AV (2.128 mg KOH/g) compared with the other lipid sources, suggesting greater susceptibility to hydrolysis. Elevated FFA levels can negatively affect sensory quality by contributing to bitterness or rancidity. Conversely, palm kernel oil, cocoa butter substitute, and sunflower oil showed low FFA and AV values, indicating good refining quality and suitability for neutral flavor applications in PCA manufacture^32^.

The absence of detectable soap content in all samples confirms effective refining and neutralization processes. Residual soaps can interfere with interfacial protein adsorption and destabilize emulsified systems; therefore, their absence supports compatibility with emulsifying salt–casein interactions during cheese analogue processing. Color measurements revealed notable differences, particularly the higher red and yellow values observed in flaxseed oil. The presence of natural pigments such as carotenoids may influence the visual appearance of cheese analogues, potentially affecting consumer perception and product standardization.

### Chemical composition of spreadable processed cheese analogue

The physicochemical properties of spreadable processed cheese analogue, as presented in Table 3, was significantly influenced by lipid source and storage time, reflecting the important role of the fat phase in regulating water retention, fat distribution, and chemical equilibrium within the protein–fat matrix. Lipid composition governs solid fat content, crystallization behavior, and lipid–protein interactions, thereby modulating matrix integrity during refrigerated storage^2^.

At fresh time, all formulations exhibited comparable moisture (61.2–61.8%) and fat contents (26.1–26.4%), confirming effective emulsification and process standardization across treatments. However, significant differences (*p* ≤ 0.05) were observed in F/DM, pH, and acidity, indicating that lipid type immediately influenced matrix structuring. Cheeses formulated with PKO and CBS showed higher F/DM (68.0–68.5%) and slightly higher pH (5.11–5.23) compared with flaxseed and sunflower oil treatments, which exhibited lower F/DM (66.8–67.4%) and higher acidity (1.90–1.95%). The higher solid fat content and crystalline network formation associated with saturated fats likely enhanced fat entrapment within the casein matrix, whereas the greater fluidity of unsaturated oils promoted increased water incorporation and dilution of dry matter^33^. Protein (3.1–3.3%) and ash (2.1–2.4%) contents remained unaffected by lipid substitution, confirming that differences were primarily related to fat–water interactions rather than protein or mineral composition.

After 30 days of storage, lipid-dependent differences became more pronounced. A significant increase in acidity and corresponding decrease in pH (*p* ≤ 0.05) were observed across all treatments. These changes were more evident in flaxseed- and sunflower-based cheeses, where acidity increased to 1.95–2.06% and pH declined to 5.05–5.10, compared with PKO- and CBS-based cheeses, which maintained lower acidity (1.85–1.96%) and higher pH (5.12–5.18). These differences indicate that storage-related biochemical reactions, including lipid oxidation and limited protein hydrolysis, progressed more rapidly in formulations rich in polyunsaturated fatty acids^34^. The extent of acidity development differed significantly among lipid sources.

**Table 3:** Physicochemical composition, functional performance, and sensory attributes of spreadable processed cheese analogue formulated with different lipid sources during refrigerated storage.

Treatment / storage		Chemical composition					Acidity / pH		Functional properties		Sensory attributes				
Treatment	Storage period (days)	Moisture (%)	Fat (%)	Protein (%)	Ash (%)	Fat/DM (%)	Acidity (%)	pH	Oil separation (%)	Meltability (%)	Appearance	Texture	Taste	Smell	Overall acceptability
**C**	**F**	61.40 ± 0.07a	26.17 ± 0.29a	3.16 ± 0.14a	2.28 ± 0.02ab	67.79 ± 0.15a	1.98 ± 0.03b	5.07 ± 0.06b	53.33 ± 0.33b	47.78 ± 0.12b	8.80 ± 0.41a	8.20 ± 0.86a	8.73 ± 0.46a	8.87 ± 0.35a	8.87 ± 0.35a
	**30 d**	61.25 ± 0.05c	26.23 ± 0.06a	3.20 ± 0.01bc	2.28 ± 0.03bc	67.69 ± 0.17c	2.04 ± 0.02b	5.03 ± 0.01e	56.99 ± 0.02b	45.39 ± 0.05c	8.40 ± 0.74a	8.00 ± 0.93a	8.53 ± 0.74a	8.73 ± 0.59a	8.67 ± 0.62a
	**60 d**	60.94 ± 0.04d	26.23 ± 0.06a	3.24 ± 0.04ab	2.34 ± 0.03b	67.16 ± 0.09bc	2.09 ± 0.02b	4.97 ± 0.01e	58.66 ± 0.05b	43.00 ± 0.05c	8.87 ± 0.35a	8.13 ± 0.83a	8.33 ± 1.05a	8.93 ± 0.26a	8.67 ± 0.72a
**T1**	**F**	61.58 ± 0.04a	26.23 ± 0.21a	3.14 ± 0.06a	2.33 ± 0.01ab	68.28 ± 0.13a	1.80 ± 0.00d	5.23 ± 0.00a	33.33 ± 0.00d	26.67 ± 0.30e	7.60 ± 0.83c	7.73 ± 0.70bc	7.07 ± 1.49c	7.53 ± 0.99bc	7.27 ± 0.88c
	**30 d**	61.42 ± 0.03b	26.25 ± 0.05a	3.16 ± 0.01c	2.32 ± 0.02b	68.03 ± 0.16ab	1.86 ± 0.01d	5.18 ± 0.01a	41.66 ± 0.02e	21.33 ± 0.04f	7.27 ± 0.80b	7.87 ± 0.83ab	6.53 ± 1.81c	7.20 ± 1.08b	7.07 ± 0.69b
	**60 d**	61.15 ± 0.04c	26.23 ± 0.06a	3.18 ± 0.04bc	2.35 ± 0.04b	67.52 ± 0.09b	1.92 ± 0.03c	5.14 ± 0.01a	46.66 ± 0.05e	18.67 ± 0.03f	7.80 ± 1.08b	8.00 ± 0.76b	7.07 ± 1.22b	7.60 ± 0.99b	7.20 ± 0.86b
**T2**	**F**	61.77 ± 0.12a	26.30 ± 0.20a	3.18 ± 0.05a	2.10 ± 0.25 cd	68.80 ± 0.30a	1.91 ± 0.03c	5.11 ± 0.01b	35.56 ± 0.92d	36.67 ± 0.03d	7.73 ± 0.80bc	7.80 ± 0.94b	7.40 ± 1.35bc	7.80 ± 0.94bc	7.47 ± 1.06bc
	**30 d**	61.53 ± 0.06a	26.27 ± 0.03a	3.20 ± 0.01b	2.11 ± 0.01d	68.27 ± 0.08a	1.96 ± 0.01c	5.08 ± 0.01c	40.89 ± 0.06f	33.01 ± 0.01e	7.53 ± 0.64b	7.47 ± 0.83b	7.27 ± 1.22b	7.73 ± 0.88b	7.33 ± 0.98b
	**60 d**	61.25 ± 0.04b	26.33 ± 0.06a	3.23 ± 0.03ab	2.16 ± 0.03d	67.96 ± 0.21a	2.06 ± 0.05b	5.03 ± 0.02c	44.44 ± 0.04f	29.33 ± 0.04e	7.93 ± 0.96b	8.07 ± 0.88b	7.60 ± 1.18b	8.13 ± 0.83b	7.67 ± 1.18b
**T3**	**F**	61.78 ± 0.15a	26.10 ± 0.10a	3.09 ± 0.11a	2.40 ± 0.04a	68.32 ± 0.20a	1.92 ± 0.03c	5.11 ± 0.01b	38.89 ± 0.22 cd	43.33 ± 0.30c	7.87 ± 0.74bc	7.80 ± 0.68b	7.20 ± 1.08bc	7.67 ± 0.72bc	7.53 ± 0.99bc
	**30 d**	61.59 ± 0.04a	26.25 ± 0.05a	3.12 ± 0.03d	2.40 ± 0.02a	68.34 ± 0.19a	1.97 ± 0.01c	5.06 ± 0.01d	46.66 ± 0.05d	36.83 ± 0.05d	7.80 ± 0.68ab	7.73 ± 0.59ab	7.00 ± 1.20b	7.53 ± 0.74b	7.33 ± 0.90b
	**60 d**	61.32 ± 0.03a	26.27 ± 0.12a	3.15 ± 0.04c	2.43 ± 0.03a	67.91 ± 0.30a	2.04 ± 0.02b	5.01 ± 0.01d	50.55 ± 0.05d	32.50 ± 0.03d	7.93 ± 1.03b	7.73 ± 1.03b	7.60 ± 1.06b	7.67 ± 0.98b	7.53 ± 0.99b
**T4**	**F**	61.36 ± 0.26a	26.17 ± 0.15a	3.10 ± 0.05a	2.22 ± 0.08bc	67.72 ± 0.25a	2.09 ± 0.02a	5.00 ± 0.01c	103.33 ± 0.17a	55.56 ± 0.12a	6.80 ± 1.37d	6.40 ± 1.18c	6.20 ± 0.41d	6.13 ± 0.74c	6.20 ± 0.56d
	**30 d**	61.12 ± 0.02d	26.23 ± 0.06a	3.16 ± 0.02c	2.24 ± 0.02c	67.47 ± 0.18c	2.14 ± 0.02a	4.96 ± 0.01f	113.66 ± 0.04a	58.33 ± 0.06a	7.07 ± 0.59c	6.80 ± 1.08c	6.67 ± 0.82c	6.27 ± 0.80c	6.53 ± 0.52c
	**60 d**	60.82 ± 0.03e	26.30 ± 0.10a	3.19 ± 0.03abc	2.25 ± 0.04c	67.12 ± 0.29c	2.25 ± 0.04a	4.90 ± 0.01f	123.99 ± 0.11a	60.00 ± 0.03a	6.87 ± 1.77c	6.40 ± 1.50c	6.13 ± 0.52c	6.40 ± 0.74c	6.33 ± 0.62c
**T5**	**F**	61.38 ± 0.23a	26.30 ± 0.20a	3.20 ± 0.08a	2.00 ± 0.00d	68.09 ± 0.80a	1.83 ± 0.03d	5.19 ± 0.05c	43.33 ± 0.30c	56.67 ± 0.00a	7.93 ± 0.96ab	8.07 ± 0.80ab	7.73 ± 1.49ab	8.13 ± 1.19ab	8.07 ± 0.88ab
	**30 d**	61.21 ± 0.03c	26.28 ± 0.03a	3.23 ± 0.02a	2.09 ± 0.07d	67.76 ± 0.09bc	1.88 ± 0.02d	5.14 ± 0.01b	47.66 ± 0.05c	57.83 ± 0.04b	7.87 ± 0.99ab	8.00 ± 0.85a	7.80 ± 1.47ab	8.13 ± 1.19ab	8.07 ± 0.88a
	**60 d**	60.92 ± 0.02d	26.23 ± 0.06a	3.25 ± 0.04a	2.03 ± 0.03e	67.13 ± 0.13c	1.95 ± 0.02c	5.11 ± 0.02b	51.13 ± 0.04c	59.50 ± 0.03b	8.07 ± 1.28b	7.80 ± 1.32b	7.67 ± 1.76b	7.53 ± 1.68b	7.53 ± 1.55b

Note: Results are expressed as mean ± SD; means with different superscripts in a column differ significantly (*p* ≤ 0.05) and among the same storage time. C= spreadable cheese analogue with butter, T1 = spreadable cheese analogue with palm kernel oil, T2 = spreadable cheese analogue with cocoa butter substitute, T3 = spreadable cheese analogue with 50%palm kernel oil + 50% cocoa butter substitute, T4 = spreadable cheese analogue with flaxseed oil, T5 = spreadable cheese analogue with sunflower oil. F=fresh time.

Cheeses formulated with flaxseed and sunflower oils showed a more pronounced increase in acidity and a greater decline in pH compared to butter-, PKO-, and CBS-based cheeses. This behavior is attributed to the higher susceptibility of polyunsaturated fatty acids to oxidative degradation, leading to the formation of free fatty acids and acidic secondary oxidation products^35^. Comparable trends have been reported in functional dairy products fortified with PUFA-rich oils, where oxidative reactions become evident even under refrigerated conditions^3^. Moisture content remained relatively stable, although unsaturated oil formulations retained slightly higher water levels (*p* ≤ 0.05), suggesting enhanced matrix flexibility and reduced fat crystallization constraints^9^. Importantly, fat and FDM values remained stable, indicating effective emulsion stabilization without oiling-off.

By 60 days, cumulative lipid-associated reactions intensified compositional differences. Acidity reached maximum values and pH declined significantly (*p* ≤ 0.05), particularly in flaxseed oil formulations, reflecting progressive oxidation and limited hydrolysis of PUFA-rich lipids^36^. Nevertheless, pH values remained within technologically acceptable limits, indicating preserved product stability. In contrast, PKO- and CBS-based cheeses exhibited superior chemical stability throughout storage, attributable to the oxidative resistance of saturated fatty acids^5^. Butter-based cheese showed intermediate behavior consistent with its mixed fatty acid profile. Moisture content showed only slight reductions at 60 days, with unsaturated oil treatments maintaining higher water retention^37^. Protein and ash contents remained stable, confirming that storage-induced modifications were predominantly lipid-driven. These findings align with previous studies emphasizing that strategic lipid selection can be used to tailor processed cheese formulations to meet both consumer health expectations and industrial performance requirements^5,38,39^.

### Meltability and oil separation

Table 3 show the meltability and oil separation of different processed samples. Meltability and oil separation are critical functional attributes of spreadable processed cheese, as they reflect the integrity of the fat–protein matrix and directly influence consumer acceptance and processing performance. From these results, both parameters were significantly affected (*p* ≤ 0.05) by fat type and storage duration, demonstrating clear formulation-dependent behavior throughout refrigerated storage. At the fresh stage, marked differences in oil separation and meltability were observed among treatments. The control cheese formulated with butter exhibited relatively high oil separation (53.33%) and moderate meltability (47.78%), reflecting the partial coalescence of milk fat globules during heating^2^. Cheeses formulated with PKO (T1) and CBS (T2) showed significantly lower oil separation (33.33% and 35.56%, respectively) and reduced meltability (26.67% and 36.67%), indicating the formation of a more rigid fat crystal network. These results are consistent with previous reports showing that saturated fats with sharp melting profiles restrict fat mobility and reduce meltability in processed cheese systems^40,41^.

In contrast, cheeses containing flaxseed oil (T4) and sunflower oil (T5) exhibited the highest meltability values (55.56% and 56.67%, respectively), accompanied by markedly increased oil separation, particularly in the flaxseed oil formulation (103.33%). The high degree of unsaturation in these oils increases lipid fluidity, weakening fat–protein interactions and facilitating fat migration upon heating. Similar findings were reported by Cunha, Grimaldi^13^, who observed enhanced meltability but increased oiling-off in processed cheeses formulated with vegetable oils rich in polyunsaturated fatty acids. The cheese formulated with butter + CBS blend (T3) showed intermediate behavior, confirming that blending fats can partially balance meltability and fat stability.

Oil separation increased significantly (*p* ≤ 0.05) in all treatments, while meltability showed variable trends depending on fat type after one month of storage. The control sample exhibited increased oil separation (56.99%) and reduced meltability (45.39%), reflecting progressive destabilization of the fat–protein matrix during storage. Similar storage-related increases in oiling-off have been attributed to ongoing fat crystallization and rearrangement within the cheese matrix^3^. PKO- and CBS-based cheeses showed moderate increases in oil separation but experienced a notable decline in meltability, particularly in T1 (21.33%), suggesting increased rigidity of the lipid phase due to further crystallization of saturated fatty acids. This behavior aligns with the findings of^42^, who reported that high solid fat content limits cheese flow and melt during heating. In contrast, flaxseed and sunflower oil cheeses maintained high meltability values (58.33% and 57.83%, respectively), although oil separation increased substantially, reaching 113.66% in the flaxseed oil formulation. These results suggest that storage accelerates lipid oxidation and structural weakening in PUFA-rich formulations, enhancing meltability but compromising fat retention. Similar trends were observed by Rafiq and Ghosh^43^, who reported increased oiling-off during storage in dairy systems enriched with omega-3 oils.

Cumulative effects became more pronounced after 60 days of storage. Oil separation reached its highest levels across all treatments, with the flaxseed oil cheese exhibiting extremely high oil separation (123.99%), followed by sunflower oil (51.13%) and the control (58.66%). This pronounced oiling-off reflects progressive fat migration and matrix breakdown over time, particularly in formulations containing unsaturated oils. Meltability further declined in saturated fat–based cheeses, reaching its lowest value in the PKO formulation (18.67%), confirming that prolonged storage exacerbates fat crystallization and matrix rigidity. Similar long-term reductions in meltability for lauric and high-melting-point fats have been documented in processed cheese analogues^42^. Conversely, flaxseed and sunflower oil cheeses maintained the highest meltability values at 60 days (60.00% and 59.50%, respectively), despite excessive oil separation. These findings highlight a clear trade-off between meltability and fat stability in PUFA-rich formulations, as also reported by Cunha, Grimaldi^13^ and Sharma, Barthwal^34^. The butter + CBS blend (T3) continued to show balanced behavior, suggesting that partial replacement strategies may mitigate excessive oiling-off while preserving functional melting characteristics.

#### Sensory evaluation

As shown in Table 3, sensory attributes of spreadable processed cheese were significantly influenced (*p* ≤ 0.05) by fat type and storage period, with clear differences observed among treatments and over refrigerated storage. Appearance scores showed significant differences (*p* ≤ 0.05) among formulations at all storage periods. The control sample consistently recorded the highest appearance scores (8.40–8.87), remaining statistically superior (*p* ≤ 0.05) to most fat-modified treatments throughout storage. This reflects the characteristic visual properties of milk-fat–based processed cheese, including uniform color and surface smoothness. Among reformulated samples, T5 and T3 exhibited relatively high appearance scores, particularly at fresh and 30-day storage, with values not significantly different from the control in some cases. In contrast, T4 showed significantly lower appearance scores (*p* ≤ 0.05) at all storage periods, likely due to increased oil separation and color instability associated with its high unsaturated fat content. Storage time did not markedly affect appearance scores within each formulation, indicating good visual stability over 60 days.

Texture scores differed significantly among treatments (*p* ≤ 0.05) and were closely aligned with instrumental texture results. The control sample maintained the highest texture scores (8.00–8.20) across all storage periods and was significantly superior (*p* ≤ 0.05) to most fat-modified samples. T5 consistently exhibited high texture scores, particularly at fresh and 30 days, and was not significantly different from the control at these stages, suggesting a well-balanced structural matrix. T1, T2, and T3 showed moderate texture scores, with no significant deterioration during storage. Conversely, T4 recorded the lowest texture scores at all storage periods, significantly lower than all other treatments (*p* ≤ 0.05), reflecting excessive softness and poor structural integrity. It was obvious that storage time had no significant negative impact on texture within individual formulations. This was in line with previous work showing that conventional dairy fats enhance structural and textural attributes compared to extensively modified or highly unsaturated fat systems, where texture is softened due to weaker protein–fat interactions. Cunha, Dias^12^, found that processed cheese analogues with 50% vegetable fat replacement were better evaluated than those with higher fat substitution ratios, indicating that moderate reformulation can preserve texture perception, while excessive unsaturated fat leads to softer textures and lower sensory scores. Sánchez-Macías, Moreno-Indias^44^, also reported that fat reduction in cheese significantly alters sensory texture and overall acceptability, highlighting the critical role of fat in mouthfeel and structure.

Taste was one of the most discriminating sensory attributes, showing significant differences among treatments at all storage periods (*p* ≤ 0.05). The control sample consistently achieved the highest taste scores (8.33–8.73), reflecting the desirable flavor profile of milk fat. T5 exhibited relatively high taste scores, particularly at fresh and 30 days, and did not differ significantly from the control in some cases. T1, T2, and T3 showed moderate taste scores, with slight but non-significant fluctuations during storage. In contrast, T4 had significantly lower taste scores (*p* ≤ 0.05) at all storage periods, likely due to flavor instability and the development of less desirable notes associated with high levels of polyunsaturated fatty acids. Storage time did not significantly affect taste scores within most treatments, indicating acceptable flavor stability over 60 days.

Smell scores followed trends similar to taste and showed significant differences among treatments (*p* ≤ 0.05). The control sample maintained the highest smell scores (8.73–8.93) throughout storage and remained significantly superior to most reformulated samples. T5 and T2 exhibited relatively high smell scores, particularly at fresh and 30 days, and were not significantly different from the control at certain storage points. T1 and T3 showed moderate smell scores, while T4 consistently recorded the lowest smell values, significantly lower than other treatments (*p* ≤ 0.05), likely due to oxidative susceptibility of its lipid fraction. The smell scores were relatively stable during storage, suggesting effective control of off-odors in most formulations. The decreased taste and smell ratings for high unsaturated fat formulations are consistent with findings by Al-Ismail, Al-Hiary^45^, who reported that the substitution of milk fat with vegetable oils significantly affected flavor in processed cheese analogues, likely due to differences in volatile compound release and oxidation products associated with polyunsaturated lipids. Furthermore, sensory literature emphasizes that the type and degree of fat replacement dramatically influence flavor perception. Studies on full-fat, reduced-fat, and low-fat cheeses indicate that flavor intensity and overall liking decline as fat content decreases or is replaced with non-dairy fats, highlighting the functional role of dairy fat in delivering flavor compounds^44^.

In addition, the overall acceptability, which integrates all sensory attributes, showed clear and statistically significant differences among treatments (*p* ≤ 0.05). The control sample consistently achieved the highest overall acceptability scores (8.67–8.87) at all storage periods and remained significantly superior to most fat-modified treatments. Among reformulated cheeses, T5 demonstrated the highest overall acceptability, particularly at fresh and 30 days, with scores not significantly different from the control, indicating strong consumer acceptance. T3 and T2 showed moderate overall acceptability, maintaining stable scores during storage without significant deterioration. In contrast, T4 exhibited the lowest overall acceptability at all storage periods, significantly lower than all other treatments (*p* ≤ 0.05), reflecting the combined negative effects of poor texture, flavor, and appearance. Although a slight decline in overall acceptability was observed with increasing storage time, these changes were generally not statistically significant within individual treatments.

This was agrees with broader reviews of plant-based or modified fat cheese products, which report that sensory acceptance frequently declines when dairy fat is extensively replaced unless counteracted with complex flavoring or structural adjustments such as hydrocolloids, and texturizers^46^. This confirms that while nutritional modification, such as reducing saturated fat or enhancing functional lipids, is beneficial, sensory acceptability remains a key challenge, particularly at extended storage times when flavor and texture changes become more apparent.

The sensory results demonstrate that fat type had a more pronounced effect on sensory quality than storage time. Traditional milk fat provided superior and stable sensory properties, while T5 emerged as the most successful fat-modified formulation, achieving high sensory scores comparable to the control for up to 30 days of storage. Conversely, formulations with high levels of polyunsaturated fats (T4) suffered from significantly reduced sensory acceptance. These findings confirm that appropriate selection and structuring of fat sources can allow the development of nutritionally improved spreadable processed cheese without compromising consumer acceptance, provided that sensory stability is maintained during storage.

### Texture profile analysis of spreadable processed cheese analogue

**Fig. 1:**
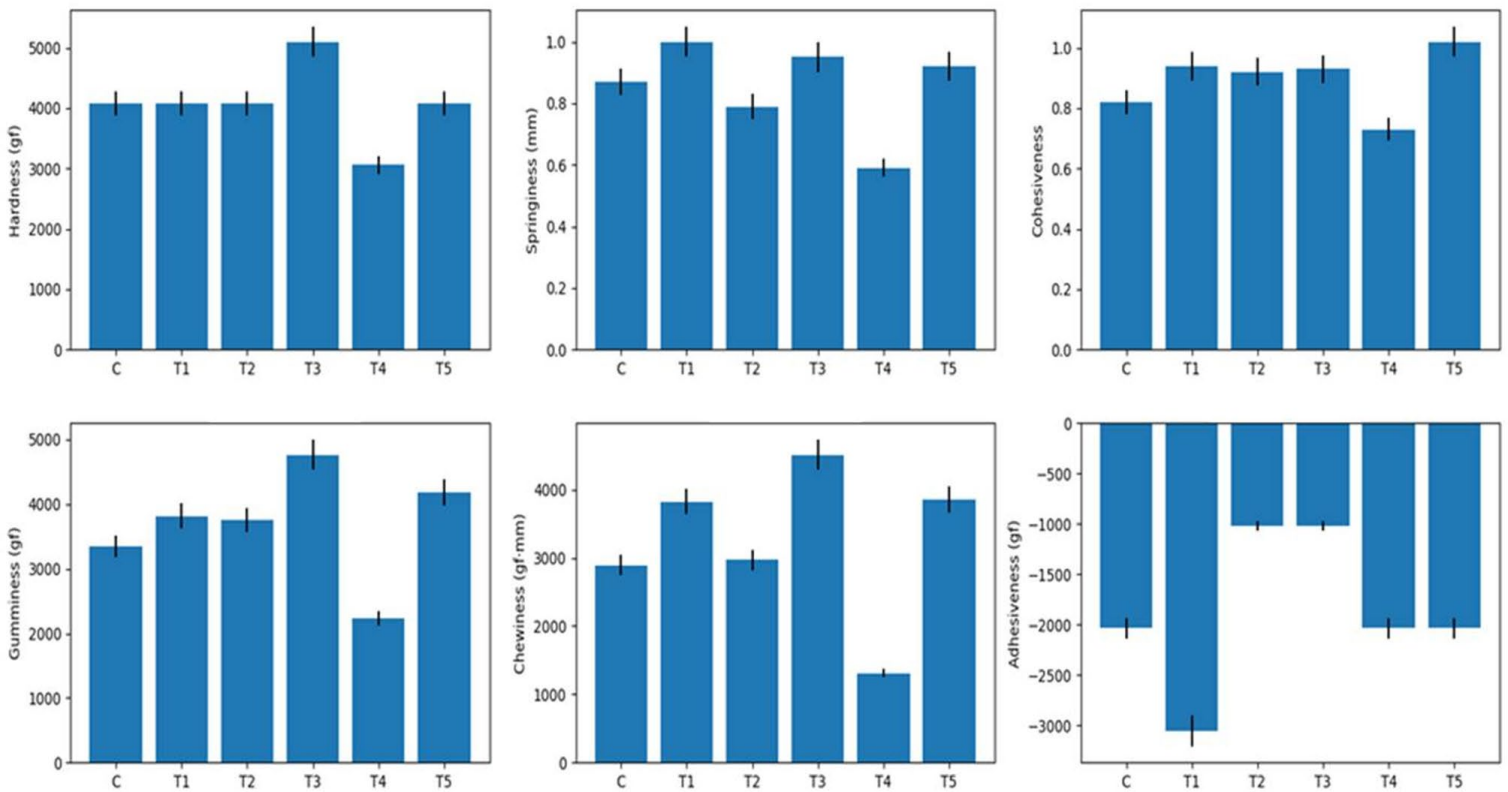
Effect of lipid source on texture profile analysis parameters of spreadable processed cheese analogue.

The functional quality of spreadable processed cheese analogue is governed by a delicate balance between mechanical strength, elastic recovery, and thermal stability, all of which are strongly influenced by the nature of the fat phase. From results, as presented in Fig. 1, a clear and mechanistically consistent relationship was established between TPA parameters, meltability, and oil separation, demonstrating that fat type plays a decisive role in defining both the structural integrity and heat-induced behavior of the cheese matrix.

Significant differences (*P* ≤ 0.05) were observed in hardness, gumminess, and chewiness among treatments, indicating substantial variation in matrix strength. Formulations exhibiting higher hardness and gumminess, particularly T3 and to a lesser extent T1 and T5, reflected the development of a dense and well-integrated protein–fat network. Such matrices resist deformation and require greater energy during compression, as confirmed by elevated chewiness values. This behavior is characteristic of systems containing fats with higher solid fat content and well-defined crystallization profiles, which reinforce the protein network and restrict molecular mobility. Similar increases in hardness and structural rigidity have been widely reported in processed cheeses formulated with saturated or structured fats^47,48^.

In contrast, the significant (*P* ≤ 0.05) lowest hardness, gumminess, and chewiness values observed in T4 indicate a weakened and less cohesive matrix. This structural softening is typically associated with the incorporation of unsaturated oils, which remain liquid at refrigeration and heating temperatures, disrupt fat crystallinity, and reduce the ability of the protein network to entrap the lipid phase^49^. Comparable weakening effects have been documented in processed cheese analogues enriched with vegetable or omega-3–rich oils^13,34^. In addition, this observation is consistent with previous findings indicating that the substitution of milk fat with unsaturated vegetable oils in processed cheese formulations generally results in compromised textural properties, particularly reduced hardness, due to the increased fluidity of the fat phase and weaker protein–fat interactions^50^. Springiness and cohesiveness provided further insight into the internal bonding and elastic recovery of the cheese matrix. Treatments exhibiting higher cohesiveness values (0.92–1.02) demonstrated stronger internal bonding and greater resistance to structural breakdown during repeated deformation^2^. This indicates effective protein–protein and protein–fat interactions, which are essential for maintaining matrix continuity. It was reported that cohesive protein networks act as physical barriers that stabilize fat droplets and limit structural collapse under stress^51^.

Conversely, the markedly (*P* ≤ 0.05) lower cohesiveness observed in T4 reflects insufficient matrix integration, rendering the structure more susceptible to deformation and collapse during heating. Such behavior is frequently reported when liquid oils replace milk fat, as unsaturated lipids interfere with protein cross-linking and weaken the continuous phase^52^. In addition, the textural differences observed were directly mirrored in meltability and oil separation behavior, revealing a strong structure–function relationship. Cheeses with higher hardness, gumminess, and cohesiveness consistently exhibited lower meltability and reduced oil separation, indicating controlled thermal flow and effective fat retention. This inverse relationship between mechanical strength and meltability has been well documented in processed cheese systems, where rigid matrices restrict melting and fat migration^53^. In contrast, formulations characterized by low hardness and cohesiveness, particularly T4, showed exceptionally high meltability accompanied by excessive oil separation. This confirms that matrix weakening facilitates uncontrolled flow upon heating, allowing the fat phase to coalesce and separate. Cunha, Grimaldi^13^ reported similar findings in cheeses enriched with polyunsaturated oils, where enhanced meltability was consistently associated with increased oiling-off due to insufficient fat entrapment within the protein network.

Adhesiveness further supported the observed trends by reflecting surface interactions and fat mobility. More negative adhesiveness values, as observed in certain treatments, indicate higher surface stickiness, often linked to localized fat migration and increased free oil at the cheese surface. Guinee^54^ noted that excessive adhesiveness in processed cheese frequently precedes oil separation during heating, particularly in systems with poorly crystallized fat phases. In contrast, treatments with lower absolute adhesiveness values exhibited improved resistance to oil separation, suggesting a more stable surface structure and balanced fat distribution. The results reveal a clear trade-off between textural strength and thermal functionality. Strong, cohesive matrices restrict meltability and oil migration but may limit desirable melting behavior, while weak and highly deformable matrices enhance meltability at the expense of fat stability. This trade-off is particularly evident in formulations containing unsaturated oils, where nutritional enhancement is accompanied by increased susceptibility to oil separation.

Importantly, treatments exhibiting intermediate behavior demonstrate that strategic fat selection or blending can partially reconcile this trade-off, achieving acceptable meltability while maintaining structural integrity and minimizing oil separation. These findings align with established mechanistic models describing fat crystallinity and protein–fat interactions in dairy matrices^40,51^.

#### Color characteristics of spreadable processed cheese analogue

Color is a critical quality attribute of spreadable processed cheese, as it directly influences consumer perception and acceptance and indirectly reflects compositional and structural changes within the cheese matrix. As shown in Table 5, significant variations (*P* ≤ 0.05) were observed in L*, a*, b*, WI, and YI among formulations containing different fat sources, highlighting the strong influence of lipid type on optical properties. The L* values ranged from 71.40 to 80.80, with the highest values recorded for T1 (80.80) and T5 (80.30), while T3 exhibited the lowest lightness (71.40). Higher L* values are typically associated with increased light scattering caused by a finely dispersed fat phase and greater moisture retention, which enhance reflectance within the cheese matrix. Similar increases in L* have been reported in processed cheeses formulated with vegetable oils and structured fats that promote uniform fat dispersion^13,47^.

**Table 4:** Color parameters (L*, a*, b*), whiteness index, and yellowness index of spreadable processed cheese analogue.

Sample	L*	a*	b*	WI (CIE)	YI (D1925/2 C)
C	79.10 ± 0.10ᶜ	-0.10 ± 0.02ᵃ	14.60 ± 0.11ᵇ	74.51 ± 0.10ᶜ	26.37 ± 0.15ᵇ
T1	80.80 ± 0.10ᵃ	-1.00 ± 0.05ᵈ	5.70 ± 0.10ᵈ	79.95 ± 0.15ᵃ	10.08 ± 0.13ᵉ
T2	74.80 ± 0.11ᵉ	-0.40 ± 0.02ᵇ	4.40 ± 0.10ᵉ	74.42 ± 0.13ᶜ	8.40 ± 0.15ᶠ
T3	71.40 ± 0.10ᶠ	-0.70 ± 0.05ᶜ	5.50 ± 0.10ᵈ	70.87 ± 0.15ᵈ	11.00 ± 0.15ᵈ
T4	75.10 ± 0.10ᵈ	-0.70 ± 0.05ᶜ	20.10 ± 0.20ᵃ	67.99 ± 0.18ᵉ	38.24 ± 0.25ᵃ
T5	80.30 ± 0.10ᵇ	-0.40 ± 0.05ᵇ	6.60 ± 0.10ᶜ	79.22 ± 0.13ᵇ	11.74 ± 0.15ᶜ

Note: Results are expressed as mean ± SD; means with different superscripts in a column differ significantly (*p* ≤ 0.05). C= spreadable cheese analogue with butter, T1 = spreadable cheese analogue with palm kernel oil, T2 = spreadable cheese analogue with cocoa butter substitute, T3 = spreadable cheese analogue with 50%palm kernel oil + 50% cocoa butter substitute, T4 = spreadable cheese analogue with flaxseed oil, T5 = spreadable cheese analogue with sunflower oil.

These trends were consistent with the WI, which varied from 67.99 (T4) to 79.95 (T1). Samples with higher WI values exhibited a brighter and more appealing white appearance, which is desirable in spreadable cheese products. The reduced WI observed in T4 suggests increased absorption of visible light, likely due to the presence of naturally pigmented unsaturated oils and enhanced yellowness. Guinee and O’Callaghan^42^ reported that oils rich in unsaturated fatty acids often reduce whiteness due to their inherent color and reduced light-scattering capacity compared with milk fat.

All samples showed slightly negative a* values (− 0.10 to − 1.00), indicating a minor shift toward the green region, which is typical for dairy-based products. The absence of large variations among treatments suggests that fat substitution had a limited effect on red–green chromaticity. Similar narrow ranges of a* values have been reported in processed cheeses formulated with different lipid systems, confirming that a* is less sensitive to fat type than L* or b*^2^. In addition, marked differences were observed in b* and YI, indicating that fat type strongly influenced yellowness. The highest b* (20.10) and YI (38.24) values were recorded for T4, while T2 exhibited the lowest b* (4.40) and YI (8.40). Elevated yellowness in T4 can be attributed to the presence of naturally colored unsaturated oil components, which intensify yellow pigmentation and reduce overall whiteness. This observation is consistent with previous reports showing that vegetable oils, particularly those rich in carotenoids or oxidizable pigments, significantly increase b* and YI values in cheese systems^13^. Conversely, formulations exhibiting lower b* and YI values displayed a more neutral and consumer-preferred appearance. Reduced yellowness has been linked to improved fat emulsification and the absence of strongly pigmented lipid fractions^2^. The results indicate that careful selection or blending of fat sources is essential to balance nutritional enhancement with visual quality, as excessive yellowness may negatively impact consumer acceptance despite potential health benefits.

#### Fatty acid profile of spreadable processed cheese analogue

The fatty acid composition of spreadable processed cheese is a critical determinant of its nutritional quality, oxidative stability, and functional behavior, particularly texture, meltability, and oil separation.

**Table 5:** Fatty acid composition and nutritional lipid quality indices of spreadable processed cheese analogue formulated with different lipid sources.

Fatty acid / Index	Notation	C	T1	T2	T3	T4	T5
Saturated fatty acids							
Butyric acid	C4:0	0.50	0.00	0.00	0.00	0.00	0.00
Caproic acid	C6:0	0.30	0.00	0.00	0.00	0.00	0.00
Caprylic acid	C8:0	0.39	0.00	0.00	0.00	0.00	0.00
Capric acid	C10:0	7.63	0.00	0.00	0.00	0.00	0.00
Lauric acid	C12:0	3.57	0.00	5.20	6.24	0.00	0.00
Myristic acid	C14:0	12.18	1.30	17.09	2.88	0.00	2.11
Pentadecanoic acid	C15:0	2.53	0.00	0.00	0.00	0.00	0.00
Palmitic acid	C16:0	22.53	39.44	29.98	34.13	10.64	12.47
Margaric acid	C17:0	5.84	0.00	0.00	0.00	0.00	0.00
Stearic acid	C18:0	9.61	11.79	23.64	15.70	4.34	5.40
**Unsaturated fatty acids**							
Palmitoleic acid	C16:1	5.26	0.00	0.00	0.00	0.00	0.00
Oleic acid	C18:1 cis	17.01	21.42	24.00	16.34	35.04	22.56
Linoleic acid	C18:2	0.00	11.83	0.00	5.43	22.32	57.52
Linolenic acid	C18:3	12.67	0.00	0.00	0.00	27.66	0.00
Trans fatty acids							
Elaidic acid	trans6–trans9 C18:1	0.00	14.22	0.09	19.28	0.00	0.00
**Lipid quality indices**							
Σ SFA		65.06	52.53	75.91	58.95	14.98	19.98
Σ UFA		34.94	33.25	24.00	21.77	85.02	80.02
Σ MUFA		22.27	21.42	24.00	16.34	35.04	22.500
Σ PUFA		12.67	11.83	0.00	5.43	49.98	57.52
USFA/SFA ratio		0.54	0.63	0.32	0.37	5.68	4.01
PUFA/MUFA		0.57	0.55	0.00	0.33	1.43	2.57
PUFA/SFA		0.19	0.23	0.00	0.09	3.34	2.88
DFA		44.55	45.04	47.64	37.47	89.36	85.42
ω6/ω3 (LA/ALA)		0.00	ND	ND	ND	0.81	ND
AI		2.14	1.34	4.314	2.38	0.13	0.26
TI		ND	3.16	ND	4.84	0.13	0.49
HPI		0.47	0.75	0.232	0.42	7.99	3.83
HH		0.78	0.816	0.459	0.50	7.99	5.49
UI		60.28	45.08	24.000	27.20	162.66	137.54
HFSA		38.27	40.74	52.270	43.25	10.64	14.58
NVI		1.18	0.84	1.589	0.94	3.70	2.24
LPS		201.51	177.73	227.82	198.85	69.93	88.70

C= spreadable cheese analogue with butter, T1 = spreadable cheese analogue with palm kernel oil, T2 = spreadable cheese analogue with cocoa butter substitute, T3 = spreadable cheese analogue with 50%palm kernel oil + 50% cocoa butter substitute, T4 = spreadable cheese analogue with flaxseed oil, T5 = spreadable cheese analogue with sunflower oil. Σ SFA = All Saturated Fatty Acids, Σ UFA = All Saturated Fatty Acids, Σ MUFA = All Monounsaturated Fatty Acids, Σ PUFA = All Polyunsaturated Fatty Acids, USFA/SFA ratio = Ratio of Unsaturated to Saturated Fatty Acids, PUFA/MUFA ratio = Ratio of Polyunsaturated to Monounsaturated Fatty Acids, PUFA/SFA ratio = Ratio of Polyunsaturated to Saturated Fatty Acids, DFA = Desirable Fatty Acids, w6/w3 (LA/ALA) ratio = Ratio of Omega-6 to Omega-3 Fatty Acids, AI = Atherogenic Index, TI = Thrombogenic Index, HPI = Health-Promoting Index, HH = Hypocholesterolemic/Hypercholesterolemic Fatty Acids Ratio, UI = Unsaturation Index, HFSA = Hypercholesterolemic Fatty Saturated Acids, NVI = Nutritive value index, LPS = Lipid preventive score.

**Fig. 2:**
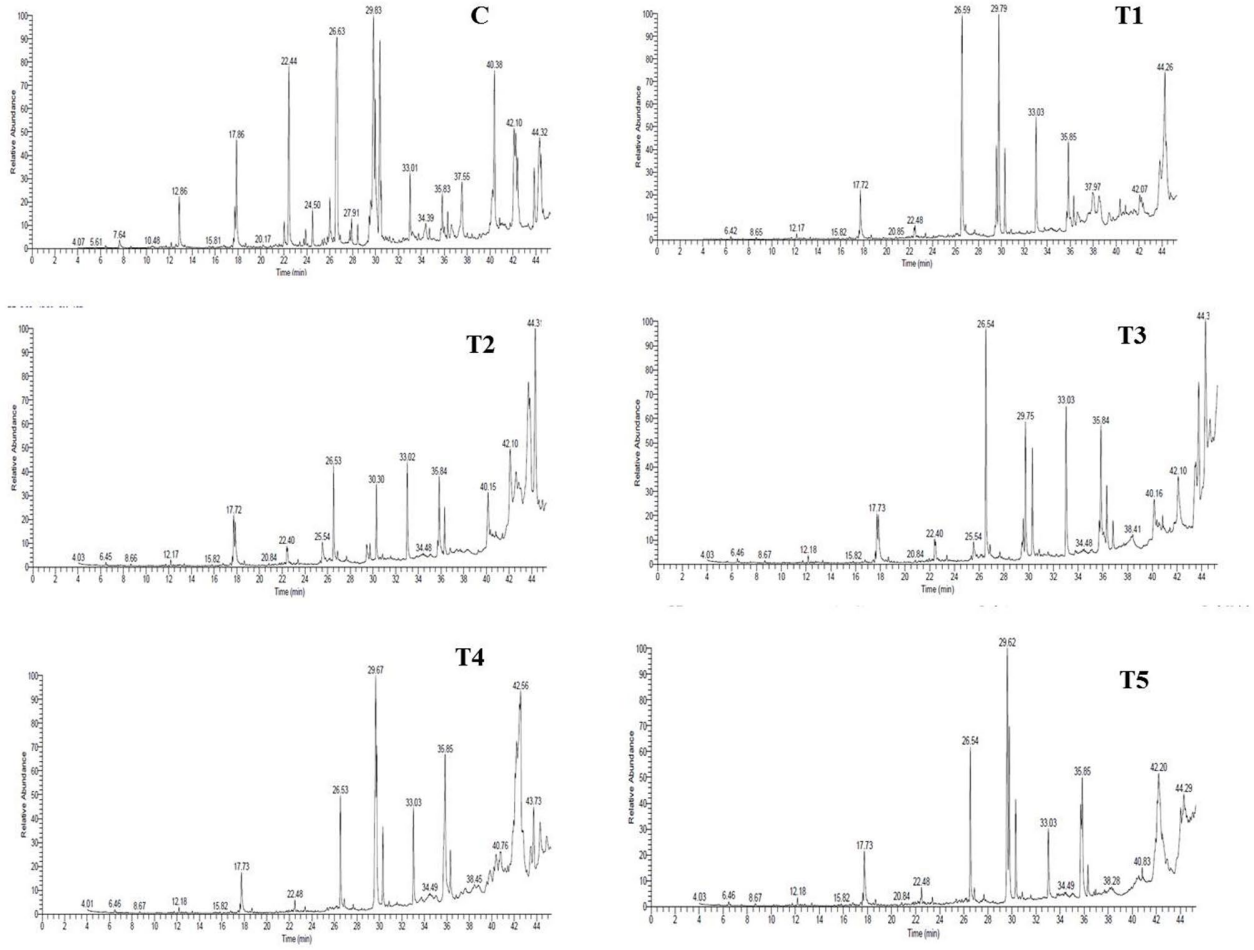
Gas chromatographic profiles of fatty acid methyl esters in spreadable processed cheese analogue as affected by lipid source.

The present results in Table 5; Fig. 2, clearly demonstrate that replacing milk fat with different lipid sources markedly altered the distribution of saturated, unsaturated, and trans fatty acids, reflecting the intrinsic fatty acid signatures of the fats used in each formulation. The control sample (C), formulated with milk fat, exhibited a typical dairy fat profile characterized by the presence of short- and medium-chain saturated fatty acids, including butyric (C4:0), caproic (C6:0), caprylic (C8:0), and capric acids (C10:0). These fatty acids are unique markers of milk fat and are absent in all vegetable-oil-based formulations, confirming the effective replacement of dairy fat in treatments T1–T5. The predominance of short-chain fatty acids in the control is consistent with well-established milk fat compositions reported in dairy literature^3^.

Palmitic acid (C16:0) and stearic acid (C18:0) were the dominant saturated fatty acids across all samples, although their relative proportions varied considerably. Treatments T1–T3 showed markedly higher palmitic acid contents (29.98–39.44%), consistent with the use of palm-based or cocoa butter substitute fats, which are known to be rich in long-chain saturated fatty acids^55^. Such fatty acid profiles promote fat crystallization and explain the higher hardness and lower meltability previously observed in these samples. Similar compositional trends have been reported for processed cheeses formulated with palm fractions and cocoa butter substitutes^10,42^.

In contrast, T4 and T5 exhibited substantially reduced total saturated fatty acid levels, reflecting the incorporation of oils with higher unsaturation. This reduction in SFAs is nutritionally advantageous and aligns with current dietary recommendations aimed at lowering saturated fat intake^56^. The unsaturated fatty acid fraction showed pronounced differences among treatments. Oleic acid (C18:1 cis) was the predominant monounsaturated fatty acid in all vegetable-oil-based samples, with particularly high levels observed in T4 (35.04%) and T5 (22.5%), consistent with oils rich in oleic acid. Increased oleic acid content is associated with improved lipid fluidity and reduced melting point, which explains the enhanced meltability and increased oil separation observed in these formulations. Comparable findings have been reported in processed cheese analogues enriched with high-oleic vegetable oils^13^.

Linoleic acid (C18:2) and linolenic acid (C18:3) were predominantly detected in T4 and T5, with T5 showing an exceptionally high linoleic acid content (57.52%) and T4 containing substantial linolenic acid (27.66%). These PUFAs significantly enhance the nutritional value of the cheese by contributing essential fatty acids and omega-3/omega-6 lipids. However, their high degree of unsaturation also increases susceptibility to oxidation and weakens fat crystallization, which mechanistically explains the lower hardness, higher meltability, and excessive oil separation observed in these treatments. Similar structure–composition relationships have been reported in functional dairy systems enriched with PUFA-rich oils^34^. In addition, Elaidic acid (trans-C18:1) was detected primarily in T1 (14.22%) and T3 (19.28%), with negligible or zero levels in the control and unsaturated-oil-based treatments. The presence of trans fatty acids is characteristic of industrially modified fats, such as partially hydrogenated oils or structured fat substitutes, and their detection confirms the processing history of the fats used in these formulations. From a nutritional standpoint, the elevated trans-fat levels in these treatments are undesirable, as trans fatty acids have been consistently linked to adverse cardiovascular health outcomes^56^. On the other hand, the absence of trans fatty acids in T4 and T5 further highlights the nutritional advantage of formulations based on natural unsaturated oils, despite their technological challenges in terms of structural stability. These results demonstrate that partial replacement strategies or structured lipid systems may offer a viable compromise, allowing for meaningful reductions in saturated and trans fatty acids while maintaining acceptable physicochemical and functional properties.

#### Lipid quality indices for spreadable processed cheese analogue

The nutritional quality of dietary fats is more accurately assessed using lipid quality indices rather than individual fatty acid percentages, as these indices integrate the combined effects of saturated, unsaturated, and bioactive fatty acids on cardiovascular health. In the ongoing results, marked differences were observed among treatments in all calculated lipid quality indices, clearly reflecting the strong influence of fat source on the atherogenicity, thrombogenicity, hypocholesterolemic potential, and overall nutritional value of spreadable processed cheese.

As presented in Table 5, the control sample and treatments T1–T3 exhibited high total saturated fatty acid (ΣSFA) contents (58.95–75.91%), typical of milk fat and palm-based or structured fats. In contrast, treatments T4 and T5 showed a dramatic reduction in ΣSFA (14.98 and 19.98%, respectively) accompanied by a substantial increase in ΣUFA (80.02–85.02%), indicating a major nutritional improvement. This shift is reflected in the USFA/SFA and PUFA/SFA ratios, which were exceptionally high in T4 (5.676 and 3.336) and T5 (4.005 and 2.879), far exceeding recommended dietary thresholds and demonstrating a lipid profile strongly associated with reduced cardiovascular risk^57^. The PUFA/MUFA ratio further highlights these differences, with T4 and T5 showing markedly higher values (1.426 and 2.556) compared with the control and saturated-fat-rich treatments. Such profiles are characteristic of oils rich in essential fatty acids and are widely associated with improved plasma lipid metabolism and anti-inflammatory effects. The AI and TI are among the most critical indicators of cardiovascular health risk. The results showed that, AI values were highest in T2 (4.314) and relatively elevated in C and T3, reflecting their high contents of atherogenic saturated fatty acids. In contrast, T4 and T5 exhibited exceptionally low AI values (0.125 and 0.261), indicating a markedly reduced atherogenic potential. A similar trend was observed for TI, where T4 (0.133) and T5 (0.499) showed substantially lower values than T1 and T3, which exhibited the highest TI values (3.160 and 4.842).

These results clearly demonstrate that replacing saturated and trans-fat-rich lipid systems with unsaturated oil sources significantly improves the cardiovascular health profile of processed cheese^58^. Such reductions in AI and TI are widely recognized as indicators of improved dietary fat quality and reduced risk of thrombosis and atherosclerosis^39^. The HH and HPI provide complementary information on cholesterol metabolism. Treatments T4 and T5 recorded the highest HPI values (7.991 and 3.827) and HH ratios (7.991 and 5.488), confirming their superior ability to promote beneficial cholesterol fractions (HDL) while reducing harmful LDL cholesterol. Conversely, the lowest HPI and HH values were observed in treatments dominated by saturated fats, particularly T2 and T3, indicating a less favorable nutritional profile. These findings strongly support the replacement of conventional dairy or palm-based fats with PUFA-rich oils to enhance the hypocholesterolemic properties of processed cheese^3^.

The UI showed a dramatic increase in T4 (162.66) and T5 (137.54), reflecting the high degree of double bonds in these formulations. While higher UI values are nutritionally advantageous, they also imply increased susceptibility to oxidation, which explains the previously observed trade-off between nutritional enhancement and oxidative or physical stability. In addition, the NVI further confirmed the superiority of T4 and T5, with values of 3.701 and 2.237, respectively, compared with values close to unity in the control and other treatments. In parallel, the LPS was lowest in T4 (69.93) and T5 (88.70), indicating a reduced lipid-related disease risk compared with saturated-fat-rich formulations, which showed LPS values exceeding 170. Moreover, DFA values were markedly higher in T4 (89.36%) and T5 (85.42%) compared with the control and saturated-fat-rich treatments (37.47–47.64%). This substantial increase reflects the dominance of unsaturated fatty acids in these formulations and indicates a lipid profile strongly associated with improved cholesterol metabolism.

High DFA values are widely recognized as nutritionally advantageous and have been linked to reduced cardiovascular risk. Conversely, HSFA values were lowest in T4 (10.64%) and T5 (14.58%), while considerably higher values were observed in the control and treatments T1–T3 (38.27–52.27%). Elevated HSFA levels are associated with increased LDL cholesterol and a higher risk of atherosclerosis, underscoring the less favorable nutritional profile of saturated- and trans-fat-rich formulations^3^. This clearly demonstrates the nutritional benefit of replacing conventional dairy or palm-based fats with unsaturated oil sources. These results are consistent with previous reports showing that increasing DFA while minimizing HSFA significantly improves the lipid health quality of dairy products and reduces their atherogenic potential^59,60^.

### Multivariate analysis of different processed cheese samples

**Fig. 3:**
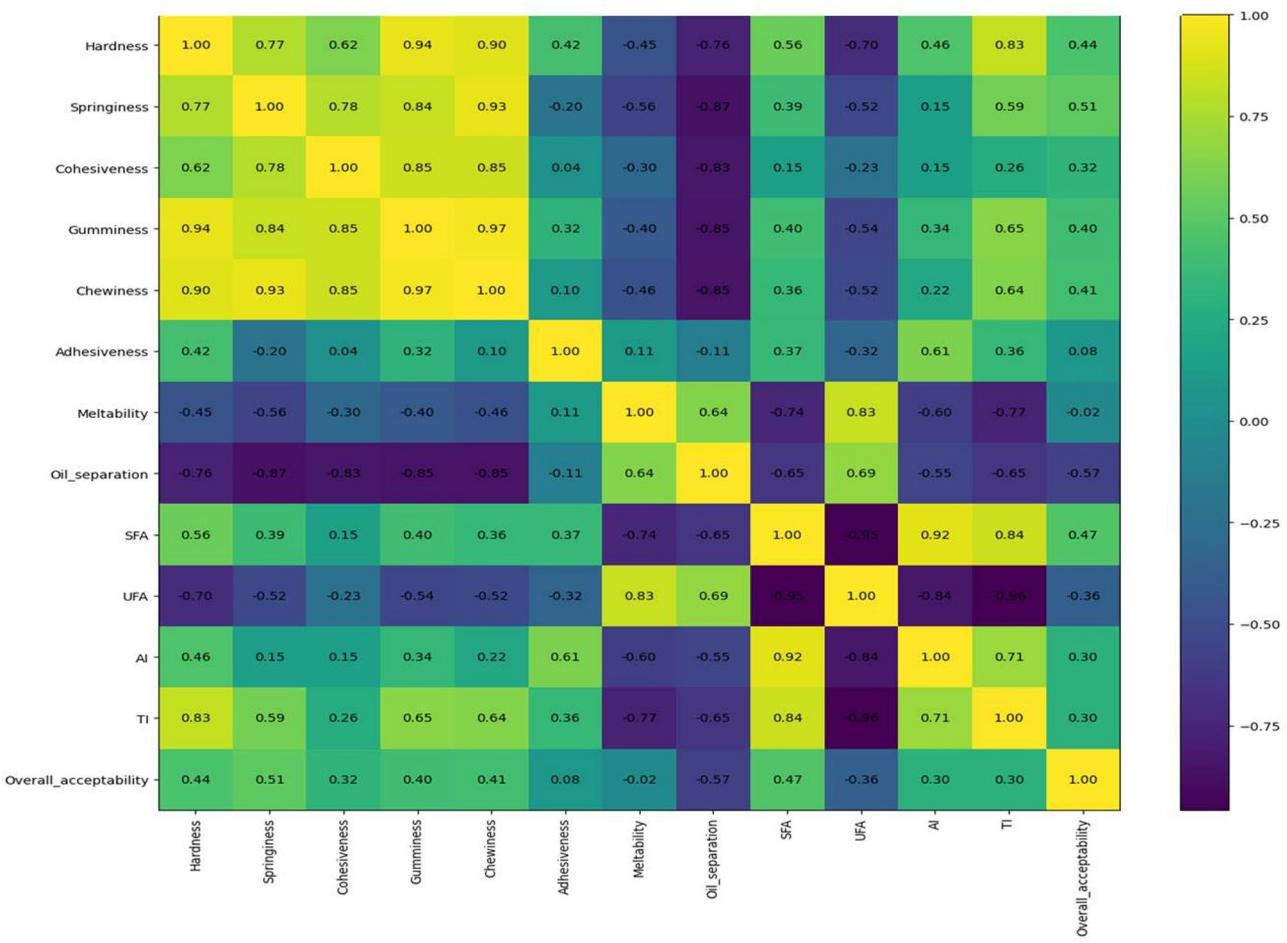
Correlation heatmap showing Pearson correlation coefficients (R) among different characteristic of spreadable processed cheese formulations.

A Pearson correlation analysis was conducted to evaluate the relationships among different characteristics of spreadable processed cheese formulations (Fig. 3). Strong positive correlations were observed among hardness, gumminess, and chewiness (*R* = 0.90–0.97), confirming their close mechanical and mathematical interdependence. This pattern indicates that fat type significantly modulated the mechanical strength and integrity of the protein–fat network, a defining feature of processed cheese texture. In addition, springiness and cohesiveness also showed positive correlations with hardness and chewiness, reflecting the development of a more elastic and cohesive matrix in samples with stronger internal bonding. These parameters are strongly influenced by protein hydration, emulsification efficiency, and fat dispersion, reinforcing the central role of fat–protein interactions in determining viscoelastic behavior. This was in line with^13,48^.

A pronounced negative correlation between texture strength (hardness, gumminess, chewiness) and meltability was evident, demonstrating that firmer cheese matrices resisted thermal softening. This relationship is characteristic of processed cheese systems, where increased protein–protein interactions and reduced fat mobility limit flow upon heating. Conversely, samples exhibiting higher meltability tended to be softer and less resistant to deformation. Moreover, oil separation exhibited strong negative correlations with all major texture parameters, confirming that weakening of the protein–fat matrix promotes fat release. The positive correlation between meltability and oil separation further indicates that excessive melting facilitates oil migration and surface fat leakage. These findings explain the technological challenges associated with highly unsaturated oil systems, where increased fluidity undermines matrix stability^13^.

The heatmap revealed a very strong inverse correlation between SFA and UFA (*R* = − 0.95), reflecting the compositional shift resulting from fat substitution strategies. SFA showed positive correlations with hardness, gumminess, and chewiness, underscoring the ability of saturated fats to crystallize and reinforce the cheese matrix. Solid or semi-solid fat fractions act as structural fillers, increasing resistance to deformation and improving shape retention. In contrast, UFA displayed strong positive correlations with meltability and oil separation, indicating that unsaturated oils increased fat mobility and weakened the protein network. This confirms that while unsaturated oils improve nutritional quality, they compromise textural strength and structural cohesion unless additional structuring mechanisms are employed^39,50^.

The AI and TI were strongly and positively correlated with SFA (AI–SFA: *R* = 0.92; TI–SFA: *R* = 0.84) and strongly negatively correlated with UFA (AI–UFA: *R* = − 0.84; TI–UFA: *R* = − 0.96). These relationships validate AI and TI as sensitive indicators of lipid nutritional quality and confirm that fat replacement with unsaturated oils substantially improves cardiovascular health indices. Interestingly, AI and TI also showed moderate positive correlations with texture strength parameters, indicating that nutritionally less favorable lipid profiles coincided with firmer, more cohesive textures. Conversely, lower AI and TI values were associated with increased meltability and oil separation, highlighting a fundamental nutrition–functionality trade-off in spreadable processed cheese formulation.

Overall acceptability demonstrated moderate positive correlations with hardness, springiness, cohesiveness, and gumminess, suggesting that panelists favored cheeses with sufficient structural integrity and elastic mouthfeel. This result emphasizes that sensory preference is closely linked to matrix stability rather than extreme softness or excessive melting. In contrast, overall acceptability showed a clear negative correlation with oil separation, confirming that visible fat leakage and greasy mouthfeel adversely affect consumer perception. Notably, acceptability exhibited only weak correlation with meltability, indicating that consumers tolerate moderate melting behavior provided oil separation is controlled and textural cohesion is maintained.

The correlation heatmap demonstrates a coherent structure–function–nutrition–sensory continuum governed primarily by fat composition. Increasing fat unsaturation markedly improves lipid health indices (lower AI and TI) but simultaneously increases meltability and oil separation, leading to weaker texture and reduced sensory acceptance. Conversely, higher saturation enhances structural stability and sensory quality but at the expense of nutritional lipid profile. These results highlight the necessity of balanced fat formulation strategies to achieve both nutritional improvement and desirable functional and sensory performance. Structural optimization approaches, such as fat blending, emulsifier optimization, or lipid structuring techniques, may be essential to mitigate the adverse technological effects associated with high unsaturation in spreadable processed cheese.

**Fig. 4:**
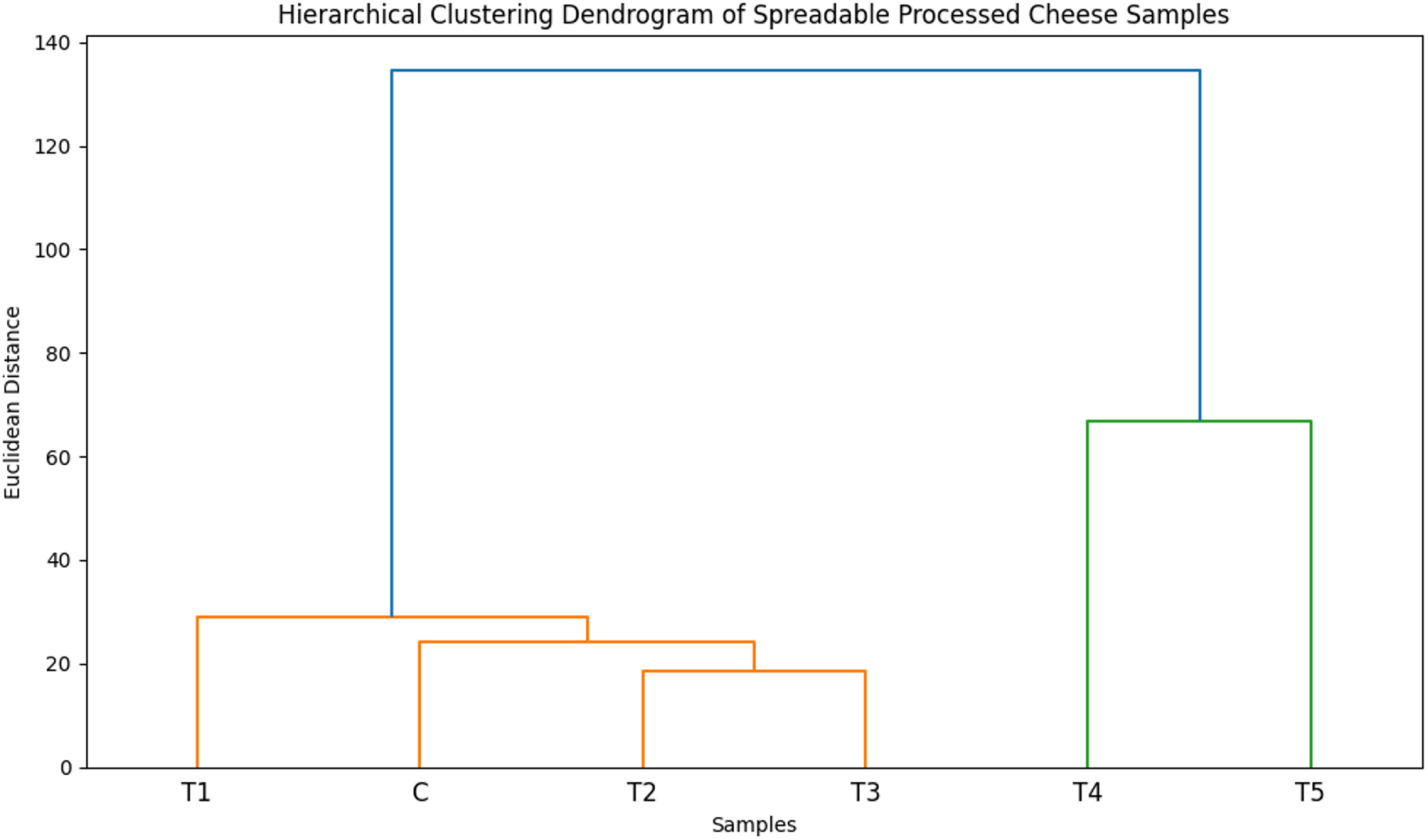
Hierarchical clustering dendrogram of spreadable processed cheese samples.

HCA was performed using Ward’s method based on Euclidean distances to explore the overall similarity among spreadable processed cheese samples formulated with different fat sources (Fig. 4). The analysis integrated physicochemical properties, functional attributes, lipid quality indices, and overall sensory acceptability, providing a holistic classification of the formulations. The dendrogram clearly separated the samples into two major clusters, indicating distinct formulation-driven behavior. The first cluster grouped the control sample together with treatments T1, T2, and T3, which are characterized by higher saturated fatty acid content, relatively lower unsaturation levels, moderate meltability, and controlled oil separation. Within this cluster, C and T1 showed the highest similarity, reflecting their comparable physicochemical stability, texture firmness, and sensory acceptance associated with more saturated fat systems. Treatments T2 and T3 formed a subcluster, indicating intermediate behavior likely resulting from partial replacement with structured or mixed fat systems.

In contrast, the second major cluster consisted of T4 and T5, which were clearly separated from the control-dominated group at a much higher Euclidean distance. This strong separation reflects the distinct lipid composition of these samples, characterized by very high unsaturated fatty acid content, low atherogenic and thrombogenic indices, increased meltability, and greater oil separation. Despite their superior nutritional lipid profiles, these samples exhibited different functional and sensory characteristics compared with the more saturated formulations. Within this cluster, T4 and T5 were closely related, although T4 showed slightly greater divergence, consistent with its extreme oil separation and lower sensory acceptability. The dendrogram confirms that fat type is the primary driver of sample differentiation, overriding minor variations in moisture, protein, and ash contents. The clustering pattern reinforces the previously observed trade-off between nutritional lipid quality and functional–sensory performance, where unsaturated oil–rich formulations form a distinct group with improved health indices but altered structural and sensory behavior. This multivariate classification supports the feasibility of designing health-oriented spreadable processed cheeses, provided that functional and sensory challenges associated with high unsaturation are appropriately managed.

**Fig. 5:**
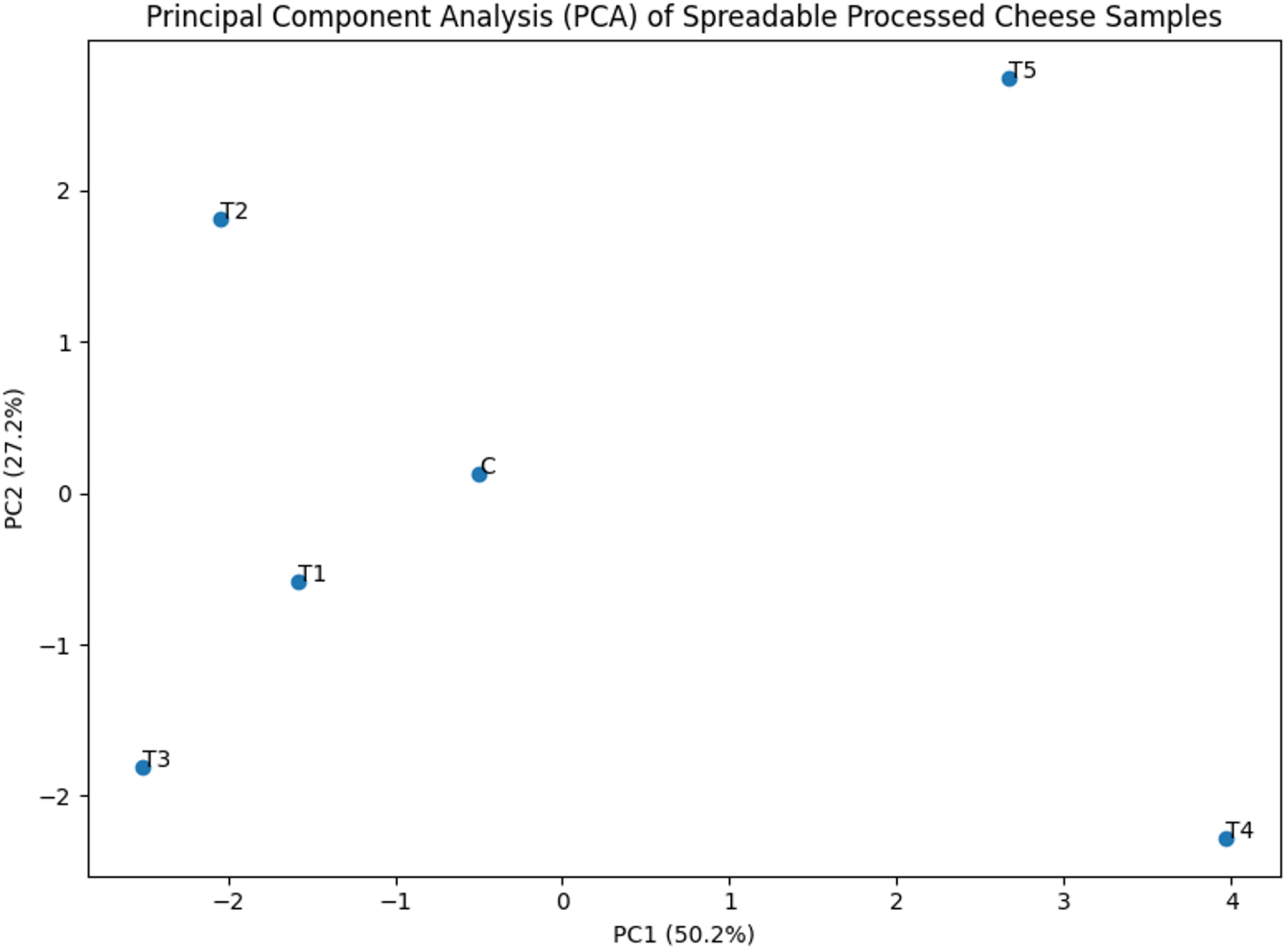
Principal component analysis (PCA) score plot of spreadable processed cheese samples.

PCA was applied to integrate physicochemical properties, functional attributes, lipid composition, health-related lipid indices, and overall sensory acceptability of the spreadable processed cheese samples. Figure 5, represent the PCA plot for different processed cheese samples.

The PC1 and PC2 explained a substantial proportion of the total variance, with PC1 accounting for 50.2% and PC2 accounting for 27.2%, representing 77.4% of the overall variability among samples. The PCA score plot clearly discriminated the cheese formulations according to fat type. PC1 primarily separated samples based on lipid composition and health indices, with positive PC1 scores associated with UFA content, lower AI and TI indices, and increased meltability and oil separation. In this regard, T4 and T5 were positioned on the positive side of PC1, indicating their strong association with unsaturated oil–rich formulations and improved nutritional lipid profiles.

Conversely, the control sample (C) and treatments T1, T2, and T3 were located on the negative side of PC1, reflecting higher SFA content, lower unsaturation levels, and more controlled functional behavior. Among these, the proximity of C and T1 indicates high similarity in overall composition, functional performance, and sensory acceptability, consistent with their clustering behavior observed in the hierarchical cluster analysis. PC2 contributed to further discrimination among the saturated and mixed-fat formulations, likely driven by variations in meltability, oil separation, and sensory attributes. Treatments T2 and T3 were clearly separated along PC2, suggesting differences in functional and sensory responses despite their relatively similar fatty acid profiles. The PCA results corroborate the findings from the correlation heatmap and dendrogram, confirming that fat type is the dominant factor governing sample differentiation. Unsaturated oil–rich formulations formed a distinct group characterized by enhanced nutritional lipid quality but altered functional and sensory properties, whereas saturated or structured fat systems clustered closer to the control with superior textural stability and sensory acceptance. This multivariate analysis highlights the intrinsic trade-off between nutritional improvement and techno-functional performance in spreadable processed cheese formulation.

## Conclusion

This study clearly demonstrates that the type of fat used in the formulation of spreadable processed cheese plays a decisive role in determining its physicochemical properties, functional performance, nutritional lipid quality, and sensory acceptance during refrigerated storage. While all formulations maintained comparable moisture, fat, and protein contents, marked differences were observed in meltability, oil separation, fatty acid composition, health-related lipid indices, and sensory attributes, highlighting the strong influence of lipid source on product behavior. Formulations rich in saturated or structured fats exhibited superior structural stability, controlled meltability, and higher sensory acceptability throughout storage, closely resembling the control sample prepared with milk fat. In contrast, unsaturated oil-rich formulations significantly improved the nutritional lipid profile, as evidenced by reduced atherogenic and thrombogenic indices and increased hypocholesterolemic potential; however, these health benefits were accompanied by increased meltability, higher oil separation, and a reduction in sensory scores, particularly after extended storage. Multivariate analyses confirmed that fat composition was the primary factor governing sample differentiation and revealed a clear trade-off between nutritional lipid quality and techno-functional and sensory performance. Samples clustered according to their degree of fat saturation, with unsaturated oil-based formulations forming a distinct group characterized by enhanced health indices but altered functional behavior.

The results indicate that it is feasible to develop nutritionally improved spreadable processed cheese by incorporating functional lipid sources, provided that formulation strategies are optimized to control oil separation and maintain desirable texture and sensory quality. Future research should focus on improving the oxidative and structural stability of unsaturated fat-based systems, potentially through fat blending, emulsifier optimization, or antioxidant incorporation, to achieve a balanced product that satisfies both health and consumer acceptance criteria.

## Data Availability

Data will be made available on request.
